# Insights into the structural dynamics of the bacteriophage T7 DNA polymerase and its complexes

**DOI:** 10.1007/s00894-018-3671-2

**Published:** 2018-06-01

**Authors:** Damian J. Magill, John W. McGrath, Vincent O’Flaherty, John P. Quinn, Leonid A. Kulakov

**Affiliations:** 1 Microbial Ecology Laboratory, Microbiology, School of Natural Sciences and Ryan Institute, National University of Ireland Galway, Galway, H91 TK33 Ireland; 2 School of Biological Sciences and Institute for Global Food Security, Medical Biology Centre, Queen’s University Belfast, 97 Lisburn Road, Belfast, BT9 7BL Northern Ireland

**Keywords:** T7 DNA polymerase, Molecular dynamics

## Abstract

The T7 DNA polymerase is dependent on the host protein thioredoxin (trx) for its processivity and fidelity. Using all-atom molecular dynamics, we demonstrate the specific interactions between trx and the T7 polymerase, and show that trx docking to its binding domain on the polymerase results in a significant level of stability and exposes a series of basic residues within the domain that interact with the phosphodiester backbone of the DNA template. We also characterize the nature of interactions between the T7 DNA polymerase and its DNA template. We show that the trx-binding domain acts as an intrinsic clamp, constraining the DNA via a two-step hinge motion, and characterize the interactions necessary for this to occur. Together, these insights provide a significantly improved understanding of the interactions responsible for highly processive DNA replication by T7 polymerase.

**Electronic supplementary material:**

The online version of this article (10.1007/s00894-018-3671-2) contains supplementary material, which is available to authorized users.

## Introduction

DNA polymerase catalyzes the synthesis of DNA—a process fundamental to the perpetuation of organisms. This is no less true with respect to dsDNA bacteriophages (phages).

T7 is one of the most studied model systems of phage structure and function [1]. The DNA polymerase of this phage (T7Pol) is an extremely efficient DNA replication apparatus [2, 3] and was discovered in T7 infected *Escherichia coli* mutants deficient in DNA polymerase I [4]. T7Pol contains a finger, palm, and thumb domain, the function of which is to position the primer-template adjacent to the nucleotide-binding site. The presence of Mg^2+^ ions via coordinate bonds with residues A476, D475, and D654, permit the formation of an octahedral coordination network with its coordination partners and water molecules, which pulls the reactive center of the reaction (the 3′-OH and nucleotide α phosphate) within close proximity and in the correct orientation. The interactions of Mg^2+^ with the 3′-OH of the primer, lowers the local pka and favors the formation of the reaction nucleophile. Together, this reduces the energetic costs of nucleophilic addition. The active site residues K522, Y526, H506, and R518 function as hydrogen bond donors and promote optimal substrate alignment for phosphoryl transfer [5]. T7Pol is able to conduct this reaction with an extremely high level of fidelity, with error rates ≤2.2 × 10^−6^ for base substitutions and ≤ 3.7 ×  10^−7^ and ≤ 4.5 ×  10^−7^ for +1 and − 1 frameshifts, respectively [6].

T7Pol alone, however, is not a very processive enzyme, and requires host derived thioredoxin (trx) as a processivity factor [7]. Stoichiometric binding (1:1) of trx to the T7Pol trx-binding domain (TBD) converts the enzyme into a highly processive DNA replication machine, which, along with the gp4 helicase, and gp2.5 ssDNA binding protein, forms the T7 replisome [8–12]. The processivity increase afforded by trx binding is well known, with studies showing how insertion of the T7Pol and T3 polymerase TBDs in *E. coli* polymerase I and *Taq* polymerase, respectively, result in increased processivity and, consequently, fidelity [13, 14]. Interestingly, despite the redox functionality of trx, this activity has been shown not to be necessary for the interaction with T7Pol [15, 16].

It has been suggested that binding of trx to the TBD results in conformational changes associated with the DNA binding groove [17], but no analysis of the dynamics of TBD association with trx has been carried out to truly determine the motions at play and the precise functionality of trx.

In this study, all-atom molecular dynamics (MD) simulations were utilized to determine the predominant modes of motion in T7Pol and its complexes, as well as the interactions occurring between T7Pol, DNA, and, critically, trx. Together, this information was used to provide a model of trx functionality in the context of the T7 DNA polymerase.

## Computational methods

### Preparation of models

The crystal structure of the T7 DNA polymerase bound to template DNA and the host protein trx (PDB code: 1skr) were obtained from the protein databank [18]. Iterative energy minimization was performed in Chimera followed by checking for steric clashes [19]. The removal of the DNA template and trx allowed the creation of four structures: T7 DNA polymerase alone (T7Pol), T7 polymerase bound to DNA (T7Pol/DNA), T7 polymerase bound to trx (T7/trx), and T7 polymerase bound to both DNA and trx (T7Pol/DNA/trx). All structures underwent energy minimization using the Yasara server, followed by checking for clashes using Chimera and the Whatif server followed by Ramachandran analysis [20]. In all cases, >97% of residues were found to lie in favored regions and no residues were discovered in forbidden regions. Following on from this, an Mg^2+^ ion was inserted in association with the co-ordination site of all models followed by another round of energy minimization and steric clash checking in Chimera.

### MD simulations

#### System set up

Hydrogen atoms were added to simulated systems according to protonation states of individual residues with protonable side chains at physiological pH. This was driven by pKa analysis using PROPKA [21].

All proteins were immersed within truncated octahedral boxes of explicit solvent (TIP3P water) with a minimum clearance of 20 Å between periodic images for starting configurations. The Amber ff99SB-ILDN force field was utilized for all simulations due to its improved side chain torsion potentials. Solvent molecules were replaced with Na^+^ and Cl^−^ ions to neutralize all charges and leave a final physiological concentration of 100 mM by using the genion facility of Gromacs v4.6.5 [22].

### Minimization, equilibration, and dynamics protocol

A round of steepest descent energy minimization was conducted with an energy step size of 0.01, until a maximum potential force of <1000 kJ mol^−1^ was achieved. The Verlet cutoff scheme was utilized, with particle mesh Ewald (PME) treatment of electrostatic interactions [23, 24]. Energy, pressure, and temperature were monitored throughout the simulation set up. Following on from this, NVT equilibration was conducted for 100 ps with an integration step of 2 fs. All bonds were constrained using the LINCS algorithm with holonomic constraints [25]. PME was implemented for long-range electrostatics with a 9 Å cutoff for the real-space term and non-bonded Van der Waals interactions were calculated using Lennard-Jones 12-6 potentials with a 9 Å cutoff. Temperature was maintained at 300 K by coupling to a modified Berendsen thermostat [26]. Velocities were derived from a Maxwell distribution. A similar set up was used for a subsequent NPT equilibration, with Parinello-Rahman pressure coupling implemented [27]. Periodic boundary conditions were used for the systems with cutoff radii of 1 nm. Systems then underwent simulation for a total time of 25 ns with integration time of 2 fs and coordinates saved every 10 ps. Independent repetitions were carried out to produce triplicates for each system under study (12 simulations in total), all of which were run on the Kelvin cluster at Queen’s University Belfast. For all properties outlined in this manuscript, replicate graphs are provided in the Supplementary Information (Figs. S1 – S16).

### Cluster analysis and analysis of interactions

Frames were extracted from the trajectory file every 10 ps, excluding the first 2 ns of the simulation, for clustering using the Gromos method within Gromacs. A neighbourhood cutoff of 3 Å was utilized for the T7Pol and a 2.5 Å cutoff for T7Pol/DNA complexes.

Electrostatic and hydrogen bond interactions were analyzed using both intrinsic Gromacs tools and the analysis facilities of VMD [28]. Confirmation of interactions was carried out by distance analysis of the residues within each cluster in PyMol [29].

### Principal component analysis

Principal component analysis (PCA) was performed to isolate the major motions occurring within the concatenated systems via diagonalization of the covariance matrix (*S*) of the positional deviation of Cα atoms with respect to an average structure. This is represented as follows:

1 $$ {S}_{ij}=\operatorname{cov}(r)=\left\langle \left({\mathrm{r}}_I-{\mathrm{r}}_{I\left(\mathrm{av}\right)}\mathrm{T}\right)\left({\mathrm{r}}_j-{\mathrm{r}}_{j\left(\mathrm{av}\right)}\mathrm{T}\right)\right\rangle \mathrm{T} $$

Whereby:

*S*_ij_: represents the element of the covariance matrix
*r*_i_: represents the coordinates of the Cα_i_ atom
*r*_j_: represents the coordinates of the Cα_j_ atom
*r*_i(av)_ and r_j(av)_: represent the coordinates of the Cα_i_ and Cα_j_ atoms of the average structures.
*T*: denotes the average time over the trajectory

Diagonalization of the matrix results in an orthogonal set of eigenvectors revealing the directions of maximum motion within the observed conformational space. This is presented as variance around the average structure. This analysis was carried out to see what the predominant modes of motion were within T7Pol.

### Affinity analysis of the T7 DNA polymerase with DNA template

In order to provide a quantitative insight into the free energy associated with biomolecular interactions, the molecular mechanics Poisson-Boltzmann surface area continuum solvation (MM/PBSA) method was utilized via the package g_mmpbsa [30]. In order to make the calculations tractable, trajectories were reduced to a 100 ps resolution. Following MM/PBSA analysis, associated Python scripts were utilized to derive estimates of per residue energy contributions to the binding process and associated plots of simulation averages produced in R.

## Results and discussion

### The predominant mode of motion is due to the TBD

**Fig. 1:**
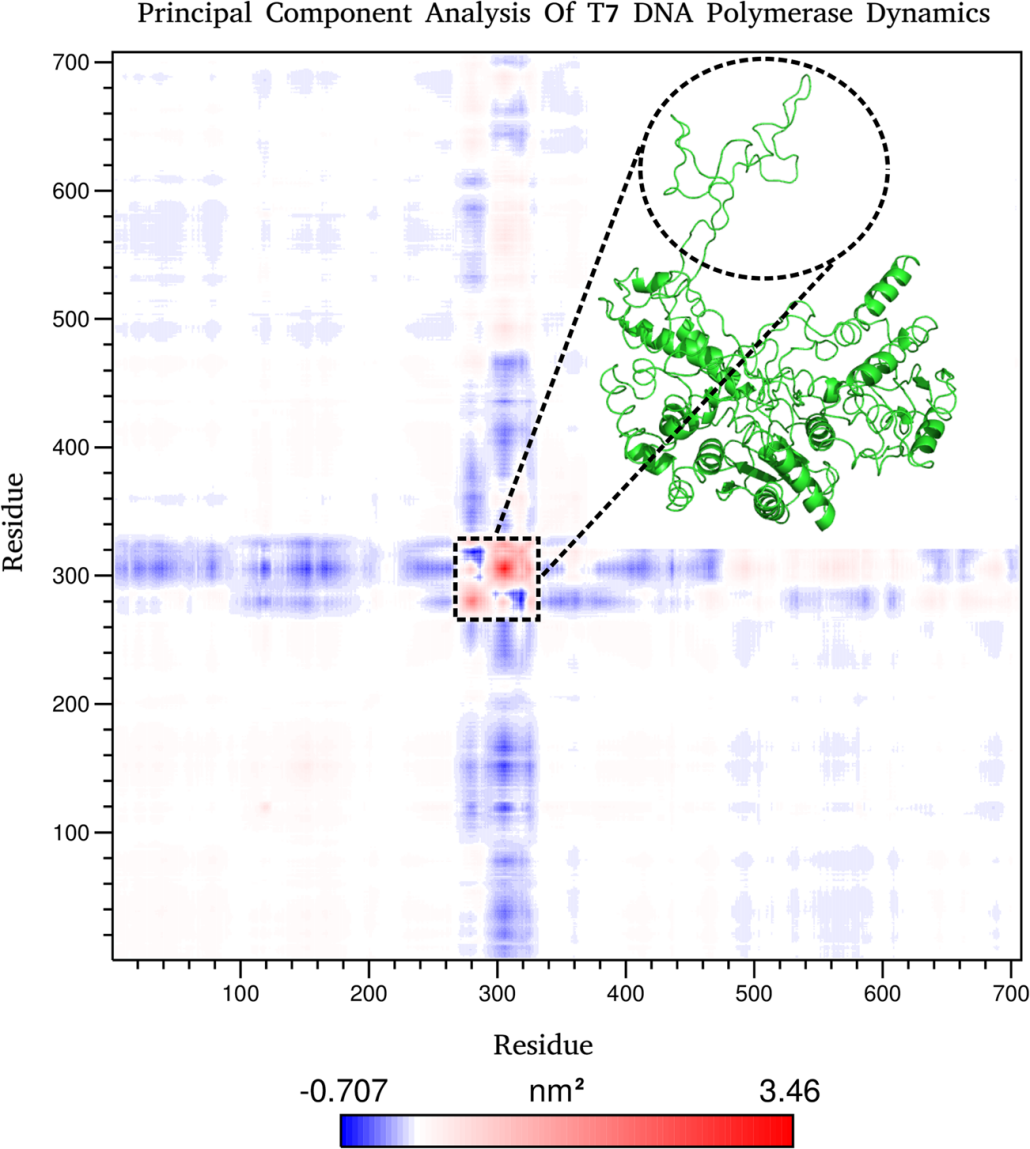
Principal component analysis (PCA) of the dominant eigenvector and root mean square fluctuation (RMSF) analysis of T7 DNA polymerase motion. PCA of the covariance matrix of T7 DNA polymerase motions determines that the major mode of motion is localized to the thioredoxin (trx)-binding domain (TBD; highlighted in* red*). RMSF shows a peak of motion around the TBD, corroborating the PCA results. Note the dip in the RMSF peak, which corresponds to a backwards loop within the TBD

**Fig. 2:**
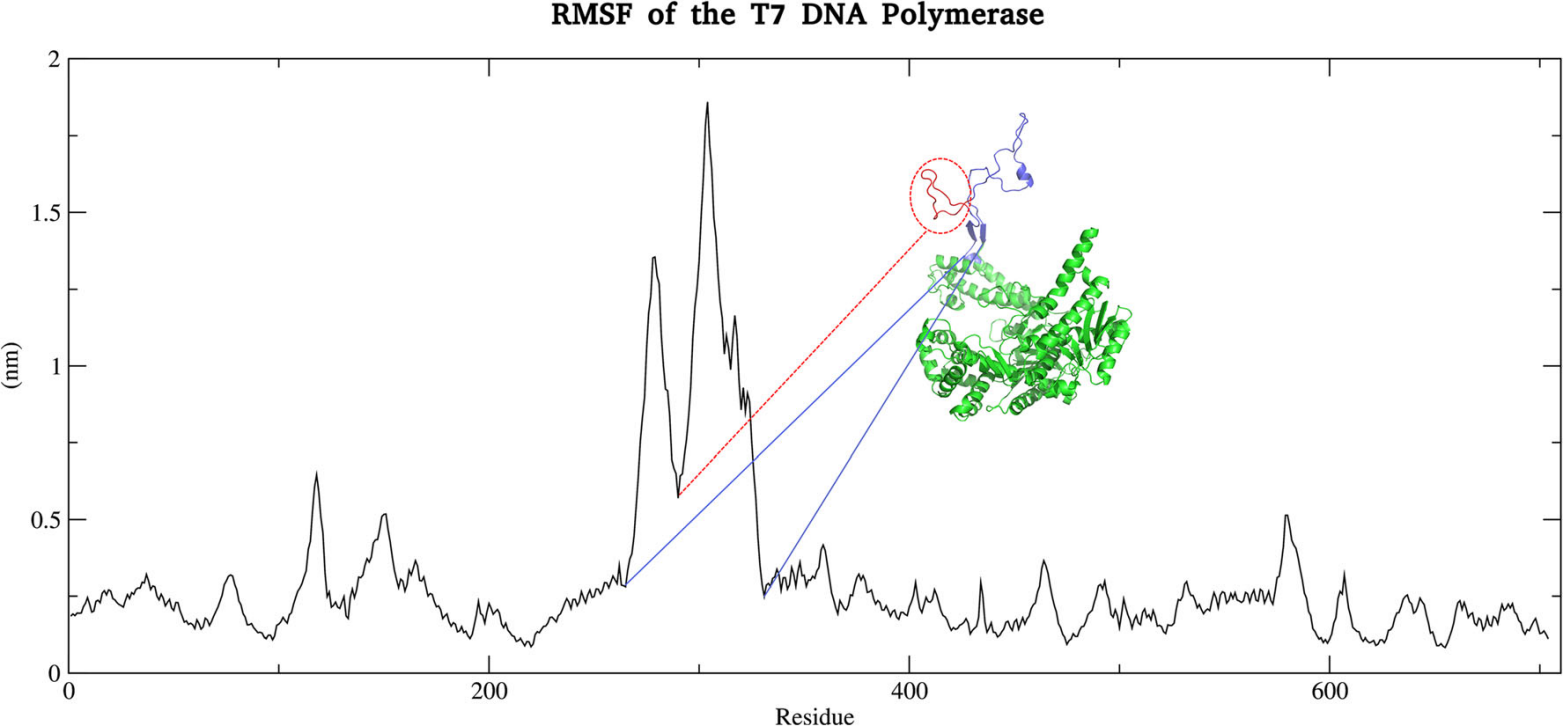
RMSF analysis of T7 DNA polymerase. Regions corresponding to the TBD and the backwards loop region of this domain are highlighted in* blue* and* red*, respectively

**Fig. 3:**
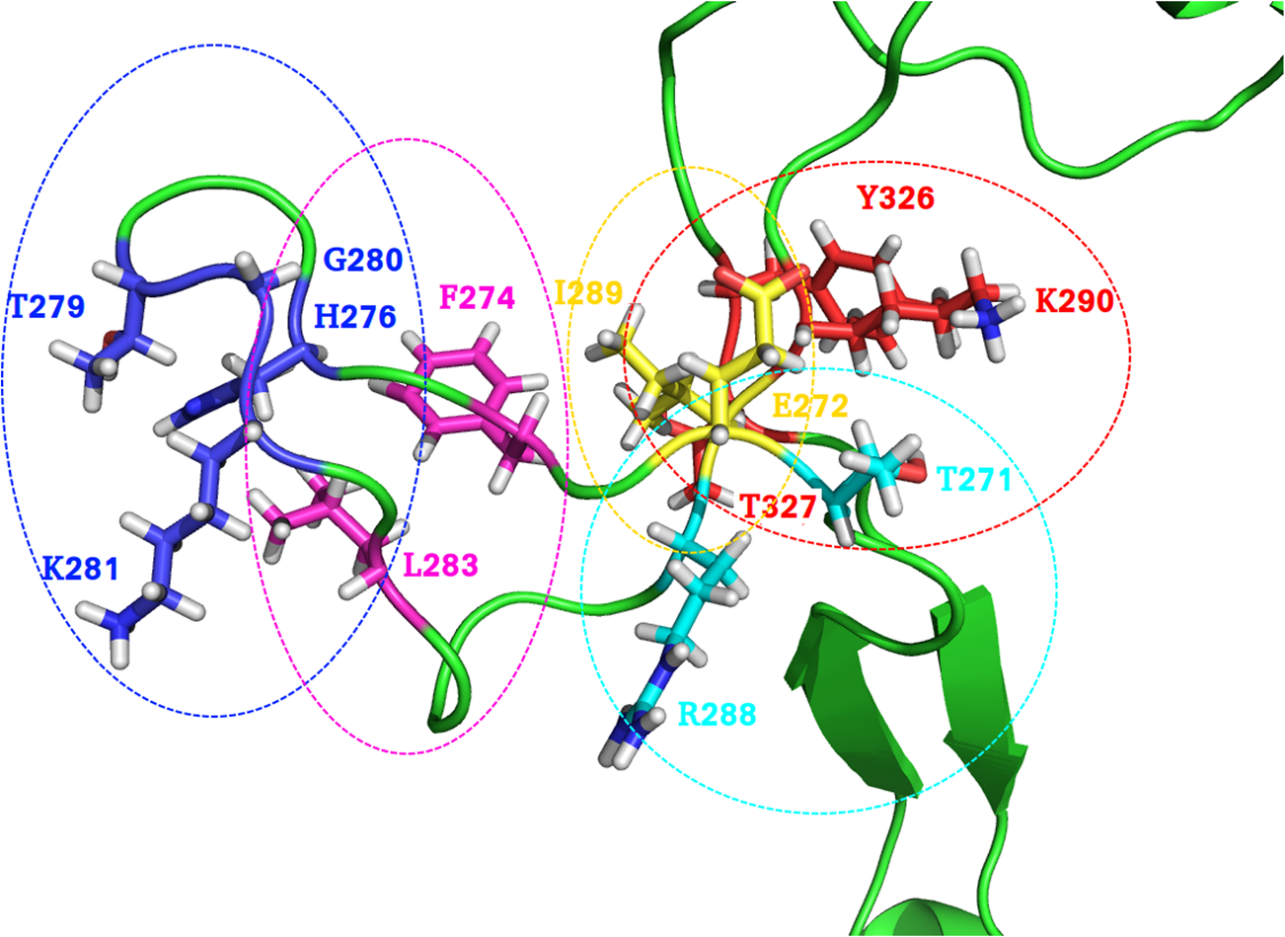
The interaction network of the backwards loop region of the T7 DNA polymerase TBD. Five networks of interaction responsible for holding together the loop are circled in varying* colors*, with corresponding residues colored similarly by element. Note that the loop is held by both distal and proximal hydrogen bonds in addition to the proposed F274/Y286 interaction

It can be seen clearly from the co-variance matrix that there is a central portion of flexibility of the T7 polymerase at approximately the 300 amino acid mark (Fig. 1). This corresponds to the TBD and is perhaps unsurprisingly revealed to be the most flexible portion of the polymerase. This is corroborated by the results of the root mean square fluctuation (RMSF) analysis, which shows a central double peak across the range of the TBD (Fig. 2) (Fig. S1). One can observe a dip in this peak (highlighted by the red line) that is not apparent from the co-variance matrix. This dip occurs for a region of amino acids from 272 to 290 corresponding to a portion of the TBD that is looped back upon itself. This loop remains intact for the duration of the simulation, showing no signs of unwinding with the overall movement of the TBD, accounting for the lower level of flexibility observed in the RMSF plot. The stability of this loop appears to be mediated in part by a series of relatively weak interactions. In this loop, a weak electrostatic contribution by residues E272 and K293 can be observed, but the major interactions maintaining the conformation of this loop are hydrogen bonds and a putative π–π interaction between F274 and Y286. Cluster analysis with a 3 Å neighbour cutoff yielded 11 clusters, with the primary cluster being occupied for ~10.2 ns of the simulation; ~87% of all conformations occupy the first 3 clusters, and here the movement of residues F274 and Y286 between sandwich, parallel displaced, and T-shaped conformations with a C4 (fourth carbon of the aromatic ring) atom distance minimum and maximum of 4.6 Å and 7.8 Å, respectively, were observed. These distances lie above the suggested limit of 3.3–3.8 Å for π–π interactions but it is likely, considering the flexible environment of these residues, that some interaction occurs here. Hydrogen bond analysis reveals 12 bonds occurring at an occupancy of >9.24%, which, all together, form five distinct interaction networks holding this backwards loop region together (Fig. 3). The importance of this loop will become apparent later when considering the role of this domain in the binding of trx/DNA.

### The TBD closes in a two-step motion

**Fig. 4:**
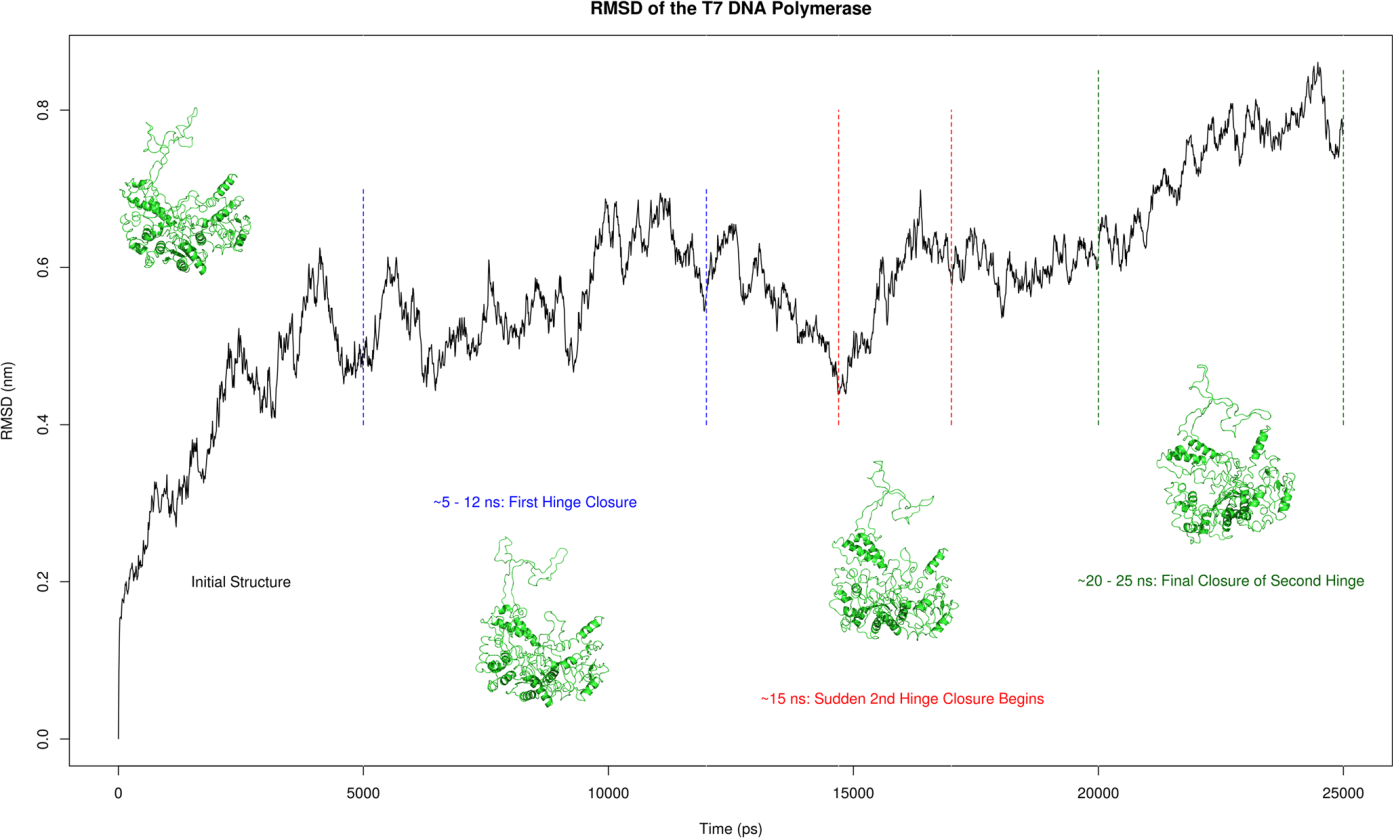
Root mean square deviation (RMSD) of T7 DNA polymerase over 25 ns all atom molecular dynamics (MD) simulation. RMSD regions corresponding to observed hinge like motions of the TBD are labelled accordingly. Representative structures at these time points are displayed showing the hinge motions of the TBD. Approximate timescales for the closure events are provided at their respective positions

Analysis of the entire 25 ns simulation of the T7 polymerase reveals a concerted movement of the TBD. This movement takes the form of a hinge-like action detailed in Fig. 4.

**Fig. 5:**
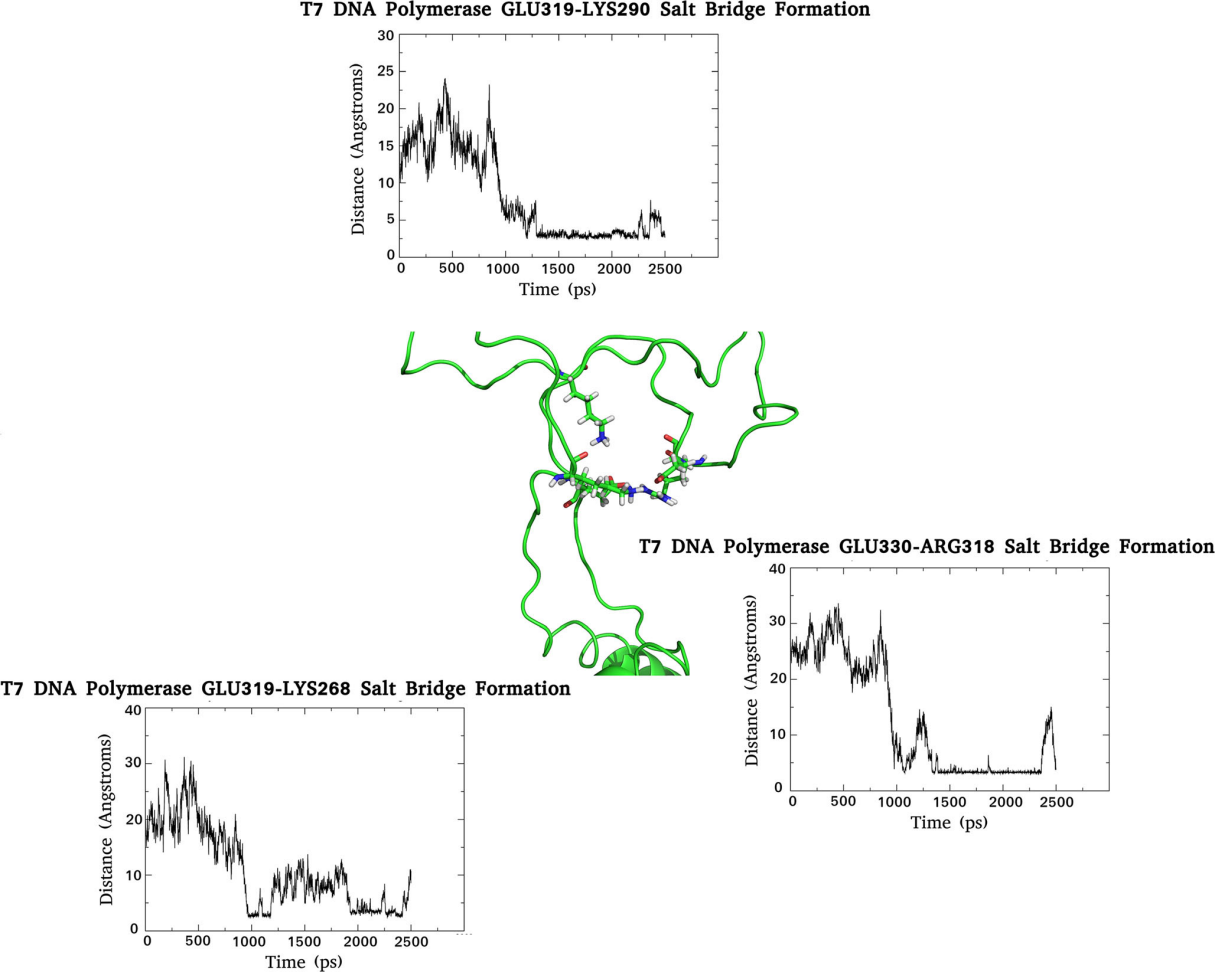
Electrostatic interactions responsible for the first hinge closure of the T7 DNA polymerase TBD. The formation of the salt bridges with time are shown in their respective graphs, and the network of interaction during the first hinge closure provided in the center of the figure. Note that the minimal plateau of each graph (representing the establishment of a stable interaction) corresponds to the time frame of the closure event

**Fig. 6:**
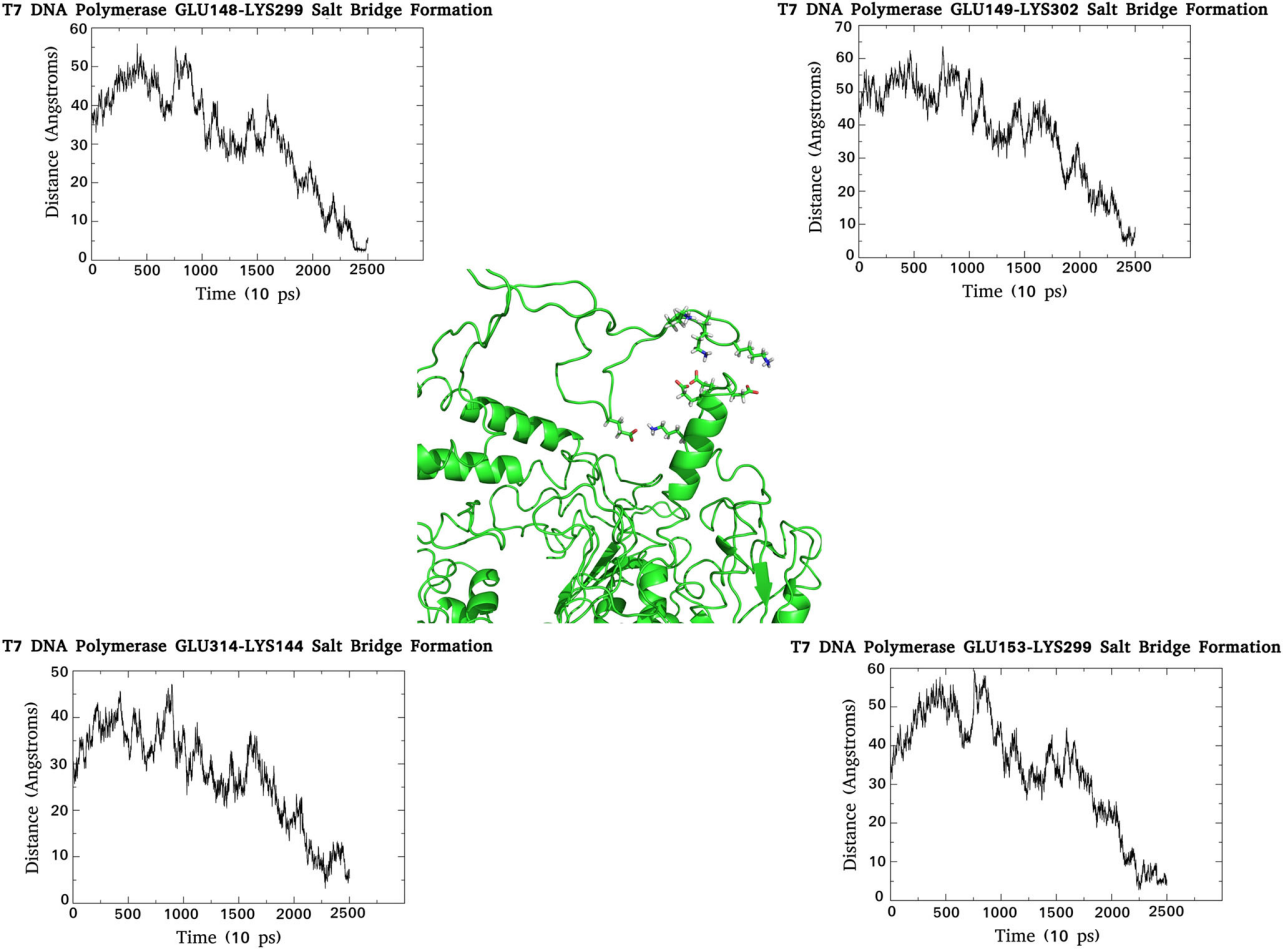
Electrostatic interactions responsible for the second hinge closure of the T7 DNA polymerase TBD. The formation of the salt bridges with time are shown in their respective graphs, and the network of interaction during the second hinge closure provided in the center of the figure. Note that the minima of each graph (representing the establishment of a stable interaction) corresponds to the time frame of the closure event

The initial 5 ns reveals little in the way of overall motion with respect to the TBD; however, from 5 ns to 12 ns, clustering of residues within the distal portion of the loop occurs and triggers the subsequent “closure” of this region. The actual closure mechanism is mediated by a series of electrostatic interactions detailed in Fig. 5 (Figs. S2 – S4). The establishment of these interactions can be observed from ~10–15 ns, correlating well with the first closure event. The critical residues involved are K290, E319, K268, E330, and R318. The K290/E319 interaction is one of those maintaining the conformation of the backwards loop region and likely represents a critical point of interaction. From 12 ns to 15 ns little change is observed but after this a sudden movement occurs in the proximal portion of the TBD that sets into motion a second closure event. This is again dominated by electrostatic interactions (Fig. 6) (Figs. S5 – S7). A number of salt bridges are observed to slowly establish between residues E148/K299, K144/E314, E149/K302, and E153/K299, reaching optimal interaction distance at the end of the simulation. These interactions likely act to both mediate the closure event and also to lock the domain in place.

Taken together, the TBD is observed to act like a clamp (not unlike that of the *E. coli* β-protein clamp), and closes in two distinct hinge-like motions, mediated by a series of electrostatic interactions. Interestingly, closure of this domain occurs in the absence of trx, which poses questions with respect to the precise role of the latter.

### DNA binding by T7Pol

**Fig. 7:**
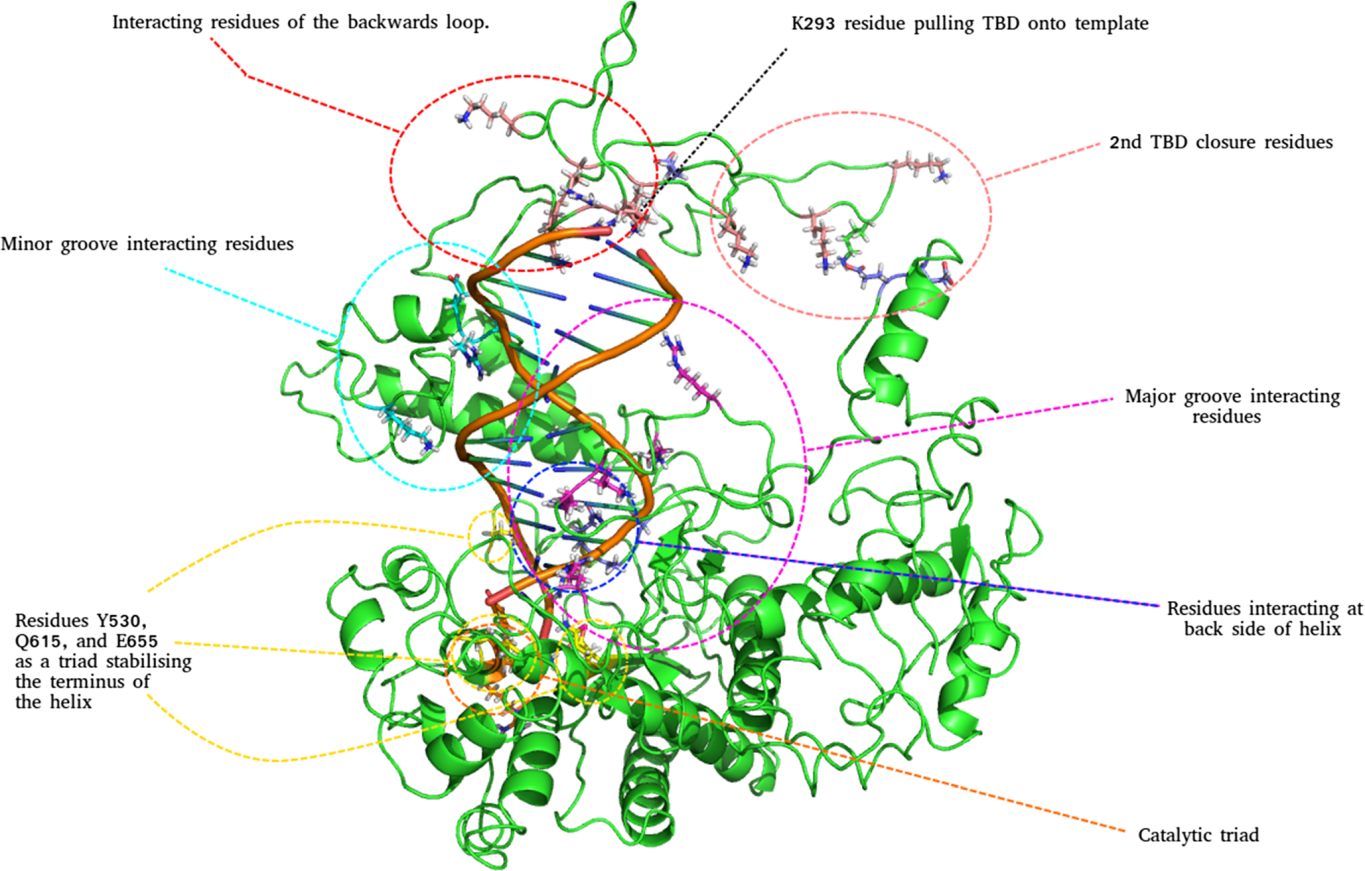
Complete network of interaction between the T7 DNA polymerase and DNA template (T7Pol/DNA). Interaction is shown at the point of second hinge closure with corresponding residues shown. Residues interacting at the major and minor grooves and behind the helix are colored and labelled accordingly. The catalytic triad and proposed terminal stabilization triad are indicated

Interactions between specific residues and DNA can be crucial in determining the processivity of a polymerase and give an insight into factors underpinning polymerase fidelity. The network of interactions between T7Pol and DNA is presented in Fig. 7.

A series of interactions surrounding the minor groove involve major contributions with the S338 side chain bonded to the DC9 O1P atom and the K355 side chain bonded to the O1P of DT20. The latter also makes electrostatic contributions with the DNA backbone. Lesser interactions are attributed to D340 bound to the DC9 O1P, similarly to its S338 neighbour, and with R339 interacting with the O3′ atom of DT8.

With respect to the major grove, the largest binding contributors are R119, which predominantly binds DG4 but also displays affinity for the O2P atom of DA18, and R604, which also shows dual binding to the DC3 O2P and DG2 O1P atoms. Other contributions are made by K404/DA5, R111/DG14, and K118 to both the O1P and O2P atoms of DG16. Again, electrostatic interactions appear to take place with adjacent lysines and the DNA backbone as an additional mode of binding. Looking outside of the grooves, interactions to the rear of the DNA involving residues N436, A438, and Q439 making contacts with the bases of DG4, DG22, and DG4, respectively, can be observed. The remainder of the contacts are made by a significant number of residues at the terminus of the DNA helix lying within the core of the protein. Residues involved here are G442, S445, and R452. G442, S445, and R452 lie higher than the others, at the side below the minor groove. These interactions however, are base specific and will show differential responses depending on the template sequence.

Interestingly, three additional residues that interact in a base-specific manner are Y530, Q615, and E655, which lie almost in a triad at the terminus of the helix. The close proximity of this “triad” to that of the catalytic site can be observed, in particular with the adjacent residues Y530 and E655. The binding provided by these residues is specifically for the DC1 and DC24 bases, i.e., the extreme terminus. These residues may act to stabilize and orientate the helix prior to its translocation to the catalytic site, which could be a crucial step with respect to the efficient function of the enzyme.

**Fig. 8:**
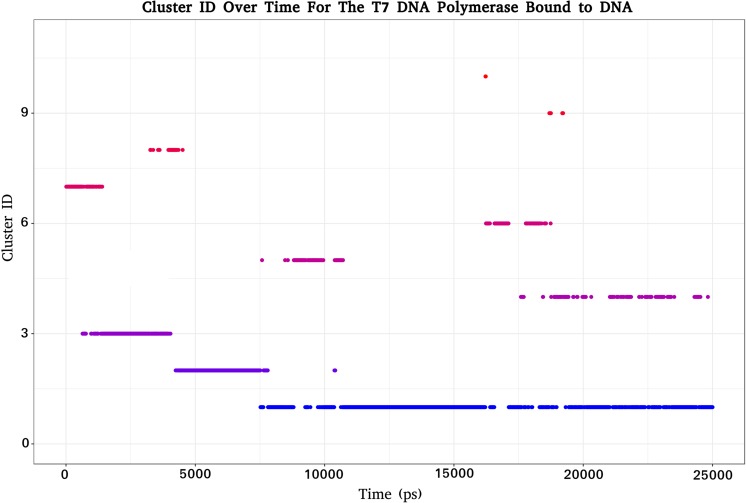
Cluster ID with respect to time for the T7Pol/DNA complex. Respective clusters are colored accordingly and displayed for the duration with which they are occupied over the course of the simulation

Cluster analysis was utilized in order to isolate preferential states of the T7Pol/DNA complex across time; 11 clusters were identified within a 2.5 Å cut-off with a total of 212 cluster transitions. A maximum of 51 transitions occur between two specific clusters. Observations of cluster identity with respect to time reveal that the primary cluster is occupied for 50.84% of the simulation time (Fig. 8). Of all the clusters, it was found that only cluster 11 represents a T7Pol/DNA complex, whereby the TBD remains in a completely open form, highlighting that some form of TBD closure occurs very early in the simulation. This is most likely due to the electrostatic interactions between positively charged residues of the TBD and the DNA backbone.

### Interactions between trx and the TBD

**Fig. 9:**
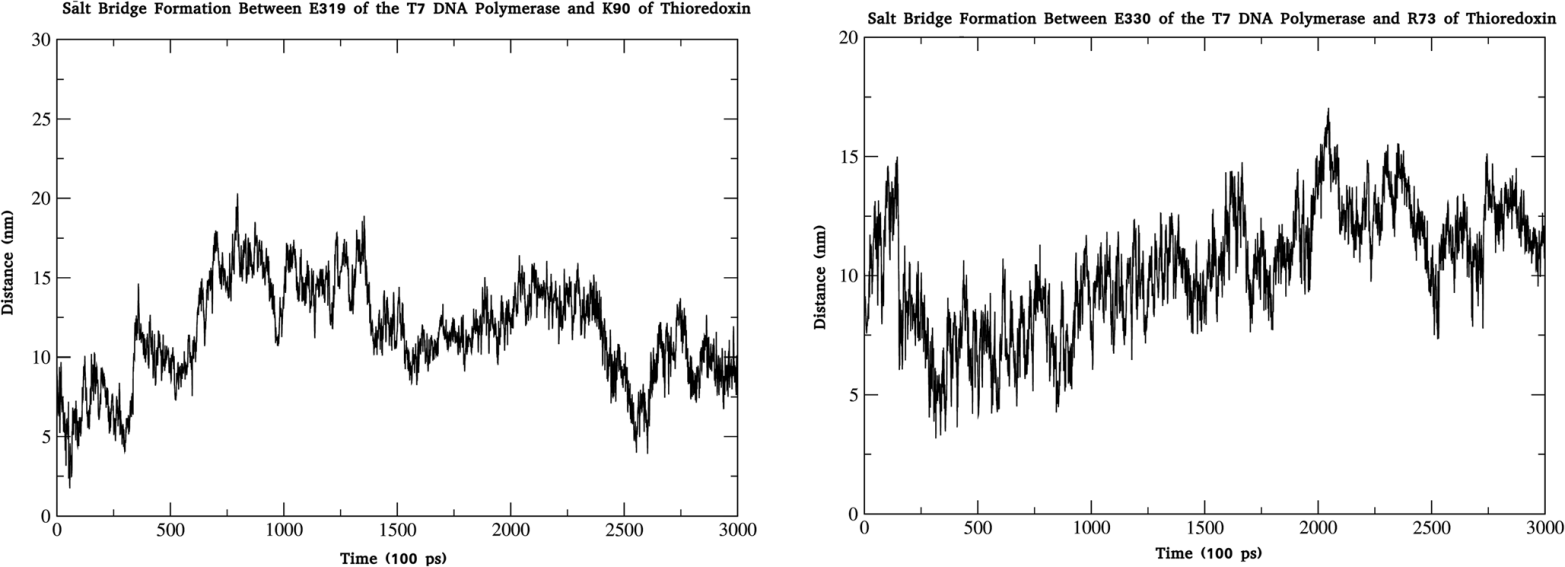
Electrostatic interactions between trx and the TBD of T7 DNA polymerase. Progression of the salt bridge interactions are shown across a 25 ns all-atom MD simulation

The host protein trx is critical for bacteriophage T7 viability. Knowledge of its interactions with the TBD facilitates elucidation of the mechanisms of fidelity in the T7 polymerase. Salt bridge analysis of T7 bound to trx revealed two interactions that take place between E319 and E330 of T7Pol, and K90 and R73 of trx, respectively (Figs. 9, S8, S9). Previous experimental work has suggested a point of interaction between trx and the TBD involving E319 of the TBD and G74 of trx [31]. A G74D mutation abolishes binding, with compensation obtained through E319K and A45T suppressor mutations. With respect to the present study, the importance of E319 is clear, but we propose that it interacts preferentially with K90 of trx. Experimental mutation of G74 to aspartate likely abolishes binding through a prohibitive electrostatic repulsion preventing the E330/R73 interaction. The E319K suppressor mutation likely acts via binding to the G74D mutation to restore function.

In regard to other interactions, I75 of trx engages in a 79% occupancy hydrogen bond with T327 of T7Pol, whilst lower frequency bonds are observed to occur between the R73/V329, I72/Y265, and A93/P325 pairs, respectively, across the duration of the simulation. It is notable that R73 engages in a separate high occupancy hydrogen bond in addition to its electrostatic contribution, highlighting the importance of this residue.

In addition to these interactions, trx likely engages in numerous hydrophobic contacts. The greatest levels of hydrophobicity are observed in the major helix and beta sheet region, both of which lie at the interface of the TBD. Taken together, these form the major points of interaction between trx and the TBD on this scale.

### Insights into the role of trx

With respect to the actual function of trx, analysis of the crystal structure of trx binding has shown that it exposes the presence of a number of basic residues that are proposed to facilitate binding [17]. This however, has never been proven from a dynamical perspective, nor has a closed TBD conformation been reported.

Here, we initially found that the motion of TBD closure is similar in the T7Pol/DNA and T7Pol/DNA/trx simulations; however, we observe that the complete closure of the domain occurs at 10–11 ns, compared to approximately twice as long in the absence of trx. This may reflect a slightly distinct mode of motion from trx-free forms of the polymerase.

**Fig. 10:**
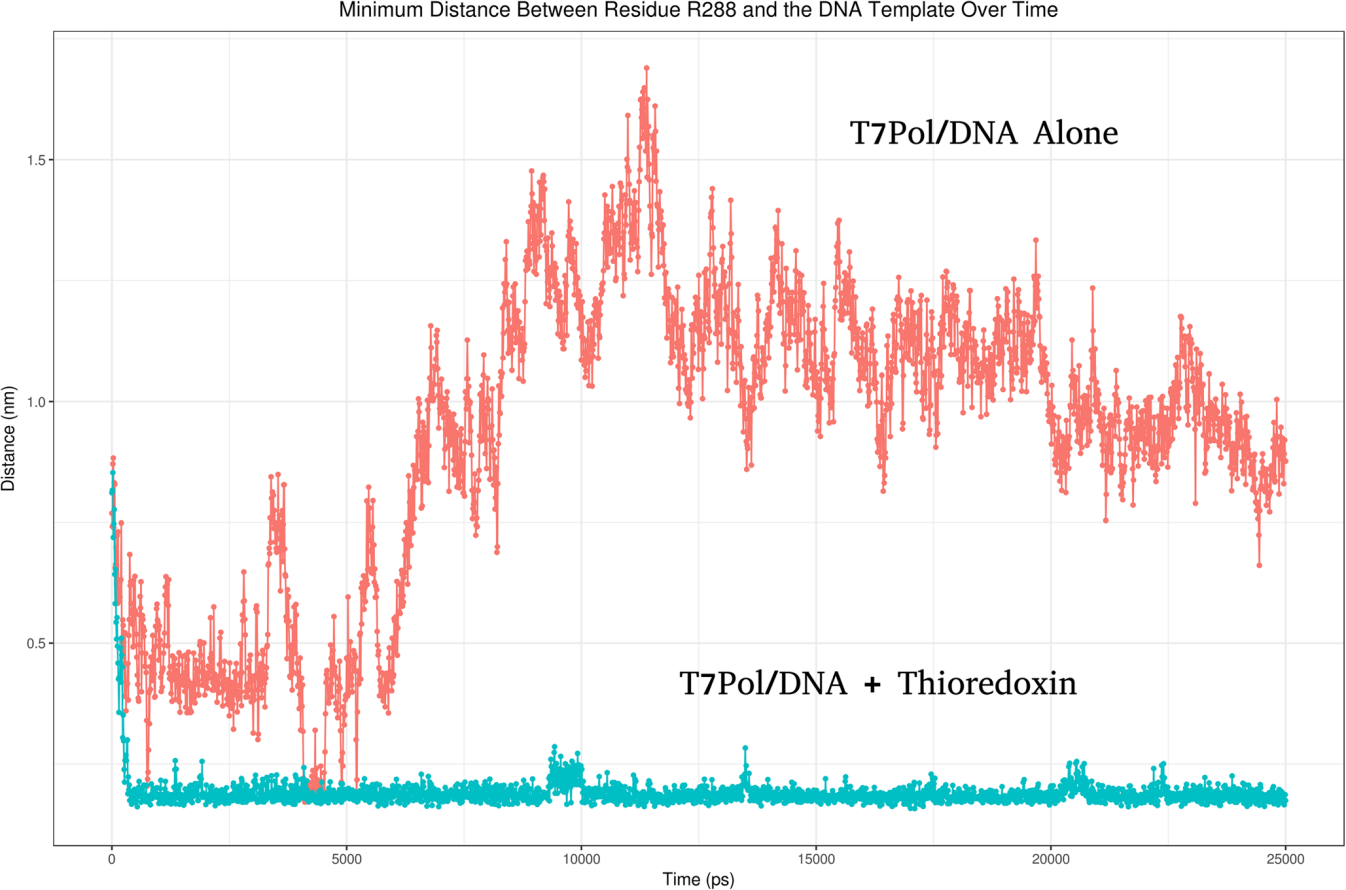
Comparison of the minimum distance of residue R288 from the DNA backbone in trx bound and unbound forms of the T7pol/DNA complex; trx bound and unbound graphs are labelled accordingly

Interactions between the TBD and trx were found to confirm previous reports that trx binding reveals a number of basic residues. We found that a series of residues were exposed that subsequently interact with the O1P and O2P atoms of DG10, DC11, and DG12. The most prominent of these residues are K281, K285, and R288. Analysis of hydrogen bonding between R288 and the DNA backbone reveal that bonding contributions are made in the T7Pol/DNA simulation, but when one analyses the minimum distances between R288 and the DNA over time in both the T7Pol/DNA and the T7Pol/DNA/trx simulation, we find a stark difference in the results, with a much greater level of interaction occurring in the latter (Fig. 10).

The additional electrostatic interactions afforded by trx binding result in a more tightly bound complex with DNA, and this likely also explains the more rapid closure time of the TBD described above. Looking globally at the DNA interactions, we also observe interactions with T271 and DG12 as well as a series of smaller contributions made by residues K268, G270, K285, K290, K300, K304, and E330. These are characterized by low occupancy hydrogen bonds and transient electrostatic interactions. Intriguingly, many of the exposed basic residues lie in the backwards loop region of the TBD which we observed to essentially lie along the helix. This region therefore seems to be crucial in determining trx functionality with respect to T7Pol.

**Fig. 11:**
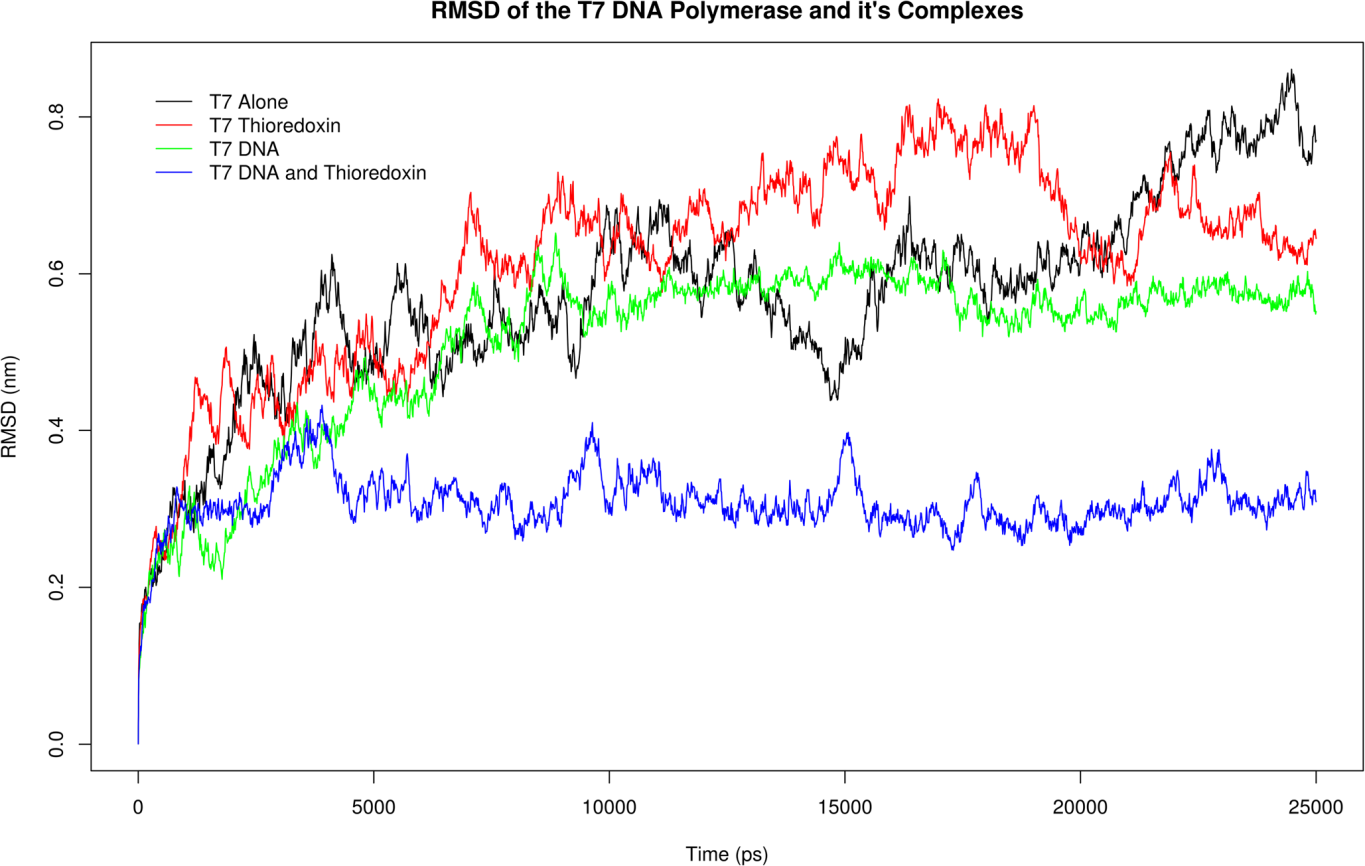
RMSD comparisons of T7 DNA polymerase and its complexes.* Colors* corresponding to each complex are provided on the plot as indicated

**Fig. 12:**
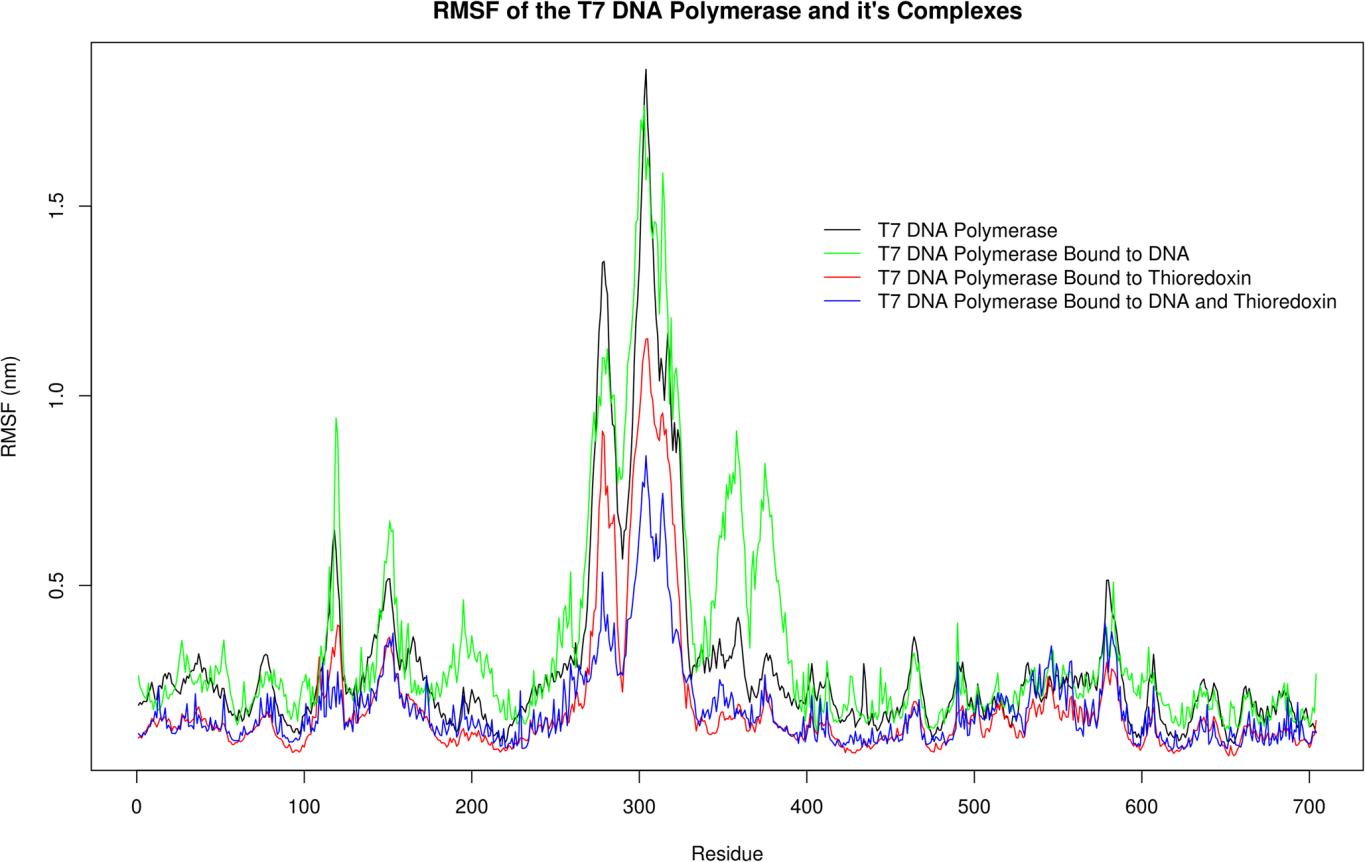
RMSF comparisons of T7 DNA Polymerase and its complexes.* Colors* corresponding to each complex are provided on the plot as indicated

Subsequent RMSD and RMSF analysis of the T7Pol complexes revealed intriguing differences between the T7Pol in all of them (Figs. 11, 12, S10–S16). Substantial differences are observed in the RMSD values between T7Pol and its DNA- and trx-bound forms (Wilcox test, V = 2,579,900, *P* < 2.2e−16; V = 918,610, p < 2.2e−16), respectively. The largest differences, however, were observed with the T7Pol/DNA/trx complex (Wilcox test, V = 3,124,900,* P* < 2.2e-16; median difference = 0.2751 Å compared with 0.0537 Å and 0.00440 Å for T7Pol/DNA and T7Pol/trx, respectively). This suggests that the most stable of the three complexes is that of the T7Pol bound to both DNA and trx. Looking at the RMSF plot, this trend is the same, but, additionally, we observe the localization of this proposed stability to the TBD as shown by lowering of the double peak region corresponding to this domain. Interestingly, the stability provided by DNA is negligible, but the presence of DNA is nevertheless necessary to achieve full stability due to trx binding. This is likely due to the increased DNA interactions facilitated by the exposed residues.

### MM/PBSA analysis of T7 DNA polymerase interactions with the DNA template

**Fig. 13:**
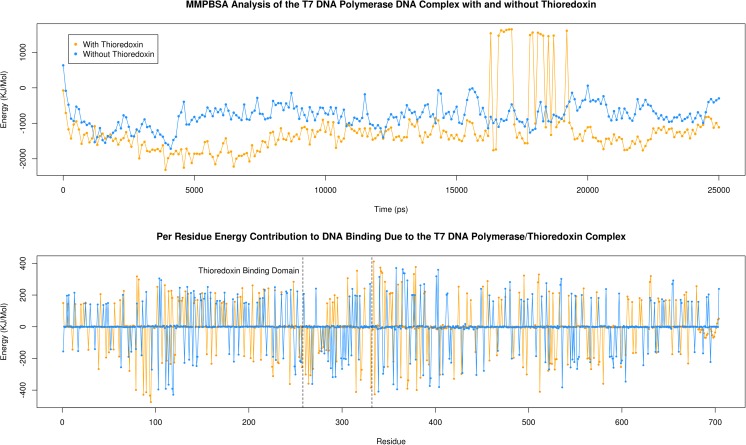
Molecular mechanics Poisson-Boltzmann surface area continuum solvation (MM/PBSA) analysis of the T7 DNA Polymerase/DNA complex and the impact of trx binding. Total average binding energy with respect to time is given for the trx bound and unbound forms in the top graph. The per residue contributions to DNA binding are given for both bound and unbound forms in the bottom graph. The region corresponding to residues of the trx binding domain is highlighted accordingly

In order to provide a quantitative insight into the binding of T7Pol for the DNA template and the impact of trx binding on this process, comparative analysis of MM/PBSA calculations was conducted. Analysis of binding energy with respect to time for the trx-bound and unbound forms, reveals a clear difference in energies between the two (Fig. 13.) with average values being −1226 kJ mol^−1^ and −778 kJ mol^−1^ respectively. The summation of the per residue energy contributions (Fig. 11) of the polymerase highlights a difference of only − 162.5 kJ mol^−1^ between the trx-bound and unbound forms. This increased capacity is likely a product of trx binding. Indeed, this disparity is found to be −891.1 kJ mol^−1^ when one considers the TBD in isolation. It seems likely that a significant proportion of the increased binding energy afforded in the trx bound form of T7Pol is due to the binding of the exposed basic residues discussed above.

## Conclusions

Trx is the only host protein required for bacteriophage T7 viability. Here, we have shown the clamp-like nature of the TBD of the T7 DNA polymerase and how the binding of trx facilitates the interaction of this domain with the DNA template, whilst stabilizing the most flexible portion of this protein. This all culminates in this intrinsic clamp binding tightly to the template, and, together with the network of interactions occurring between the T7 polymerase and DNA, forming a tightly bound complex, which is reflected in the high processivity and fidelity of this enzyme. This supports single molecule fluorescence experiments that highlighted the fact that trx binding suppresses microscopic hopping on and off from the DNA template [32]

The contributions made by trx are not limited to those seen here however. It is known that interactions with the helicase are also crucial in achieving high processivity [10]. Therefore, additional research is necessary into the entire T7 replisome to elucidate the full basis of fidelity. Here, however, we have provided one element of this understanding.

## Electronic supplementary material

ESM 1 (PNG 269 kb)

ESM 2 (PNG 626 kb)

ESM 3 (PNG 676 kb)

ESM 4 (PNG 631 kb)

ESM 5 (PNG 613 kb)

ESM 6 (PNG 576 kb)

ESM 7 (PNG 523 kb)

ESM 8 (PNG 904 kb)

ESM 9 (PNG 786 kb)

ESM 10 (PNG 311 kb)

ESM 11 (PNG 301 kb)

ESM 12 (PNG 301 kb)

ESM 13 (PNG 303 kb)

ESM 14 (PNG 290 kb)

ESM 15 (PNG 322 kb)

ESM 16 (PNG 256 kb)

## References

[1] Demerec M, Fano U (1945). Bacteriophage-resistant mutants in* Escherichia coli*. Genetics.

[2] Richardson CC (1983) Replication of bacteriophage T7 DNA. In: Becker Y (ed) Replication of viral and cellular genomes. Nijhoff, The Hague, pp 163–204

[3] Hamdan SM, Richardson CC (2009). Motors, switches, and contacts in the replisome. Annu Rev Biochem.

[4] Grippo P, Richardson CC (1971). Deoxyribonucleic acid polymerase of bacteriophage T7. J Biol Chem.

[5] Doublié S, Ellenberger T (1998). The mechanism of action of T7 DNA polymerase. Curr Opin Struct Biol.

[6] Kunkel TA, Patel SS, Johnson KA (1994). Error-prone replication of repeated DNA sequences by T7 DNA polymerase in the absence of its processivity subunit. Proc Natl Acad Sci USA.

[7] Tabor S, Huber HE, Richardson CC (1987). *Escherichia coli* thioredoxin confers processivity on the DNA polymerase activity of the gene 5 protein of bacteriophage T7. J Biol Chem.

[8] Ghosh S, Hamdan SM, Cook TE, Richardson CC (2008). Interactions of *Escherichia coli* thioredoxin, the processivity factor, with bacteriophage T7 DNA polymerase and helicase. J Biol Chem.

[9] Hamdan SM, Marintcheva B, Cook T, Lee SJ, Tabor S, Richardson CC (2005). A unique loop in T7 DNA polymerase mediates the binding of helicase-primase, DNA binding protein, and processivity factor. Proc Natl Acad Sci USA.

[10] Hamdan SM, Johnson DE, Tanner NA, Lee JB, Qimron U, Tabor S, van Oijen AM, Richardson CC (2007). Dynamic DNA helicase-DNA polymerase interactions assure processive replication fork movement. Mol Cell.

[11] Mark DF, Richardson CC (1976)* Escherichia coli* thioredoxin: a subunit of bacteriophage T7 DNA polymerase. Proc Natl Acad Sci USA 73:780–784 10.1073/pnas.73.3.780 PMC336002 768986

[12] Modrich P, Richardson CC (1975). Bacteriophage T7 deoxyribonucleic acid replication *in vitro*. Bacteriophage T7 DNA polymerase: an emzyme composed of phage-and host-specific subunits. J Biol Chem.

[13] Bedford E, Tabor S, Richardson CC (1997). The thioredoxin binding domain of bacteriophage T7 DNA polymerase confers processivity on *Escherichia coli* DNA polymerase I. Proc Natl Acad Sci USA.

[14] Davidson JF, Fox R, Harris DD, Lyons-Abbott S, Loeb LA (2003). Insertion of the T3 DNA polymerase thioredoxin binding domain enhances the processivity and fidelity of Taq DNA polymerase. Nucleic Acids Res.

[15] Huber HE, Russel M, Model P, Richardson CC (1986). Interaction of mutant thioredoxins of *Escherichia coli* with the gene 5 protein of phage T7. The redox capacity of thioredoxin is not required for stimulation of DNA polymerase activity. J Biol Chem.

[16] Huber HE, Tabor S, Richardson CC (1987). *Escherichia coli* thioredoxin stabilizes complexes of bacteriophage T7 DNA polymerase and primed templates. J Biol Chem.

[17] Akabayov B, Akabayov SR, Lee SJ, Tabor S, Kulczyk AW, Richardson CC (2010). Conformational dynamics of bacteriophage T7 DNA polymerase and its processivity factor, *Escherichia coli* thioredoxin. Proc Natl Acad Sci USA.

[18] Doublie S, Tabor S, Long AM, Richardson CC, Ellenberger T (1998). Crystal structure of a bacteriophage T7 DNA replication complex at 2.2 Ångstrom resolution. Nature.

[19] Pettersen EF, Goddard TD, Huang CC, Couch GS, Greenblatt DM, Meng EC, Ferrin TE (2004). UCSF chimera—a visualization system for exploratory research and analysis. J Comput Chem.

[20] Krieger E, Joo K, Lee J, Lee J, Raman S, Thompson J, Tyka M, Baker D, Karplus K (2009). Improving physical realism, stereochemistry, and side-chain accuracy in homology modeling: four approaches that performed well in CASP8. Proteins: Struct Funct Bioinf.

[21] Rostkowski M, Olsson MH, Søndergaard CR, Jensen JH (2011). Graphical analysis of pH-dependent properties of proteins predicted using PROPKA. BMC Struct Biol.

[22] Hess B, Kutzner C, Van Der Spoel D, Lindahl E (2008). GROMACS 4: algorithms for highly efficient, load-balanced, and scalable molecular simulation. J Chem Theory Comput.

[23] Darden T, York D, Pedersen L (1993). Particle mesh Ewald: an N· log (N) method for Ewald sums in large systems. J Chem Phys.

[24] Grubmüller H, Heller H, Windemuth A, Schulten K (1991). Generalized Verlet algorithm for efficient molecular dynamics simulations with long-range interactions. Mol Simul.

[25] Hess B, Bekker H, Berendsen HJ, Fraaije JG (1997). LINCS: a linear constraint solver for molecular simulations. J Comput Chem.

[26] Berendsen HJ, Postma JV, van Gunsteren WF, DiNola ARHJ, Haak JR (1984). Molecular dynamics with coupling to an external bath. J Chem Phys.

[27] Parrinello M, Rahman A (1980). Crystal structure and pair potentials: a molecular-dynamics study. Phys Rev Lett.

[28] Humphrey W, Dalke A, Schulten K (1996). VMD: visual molecular dynamics. J Mol Graph.

[29] DeLano WL (2002) The Pymol molecular graphics system. Delano Scientific, San Carlos, CA

[30] Kumari R, Kumar R, Lynn A, Open Source Drug Discovery Consortium (2014). g_mmpbsa: a GROMACS tool for high-throughput MM-PBSA calculations. J Chem Inf Model.

[31] Himawan JS, Richardson CC (1996). Amino acid residues critical for the interaction between bacteriophage T7 DNA polymerase and *Escherichia coli* thioredoxin. J Biol Chem.

[32] Etson CM, Hamdan SM, Richardson CC, van Oijen AM (2010). Thioredoxin suppresses microscopic hopping of T7 DNA polymerase on duplex DNA. Proc Natl Acad Sci USA.

