# Supplementary figures and images for: Insights into the structural dynamics of the bacteriophage T7 DNA polymerase and its complexes
Source: J Mol Model. 2018 Jun 1;24(7):144. doi: 10.1007/s00894-018-3671-2 (PMC5984650; doi:10.1007/s00894-018-3671-2)

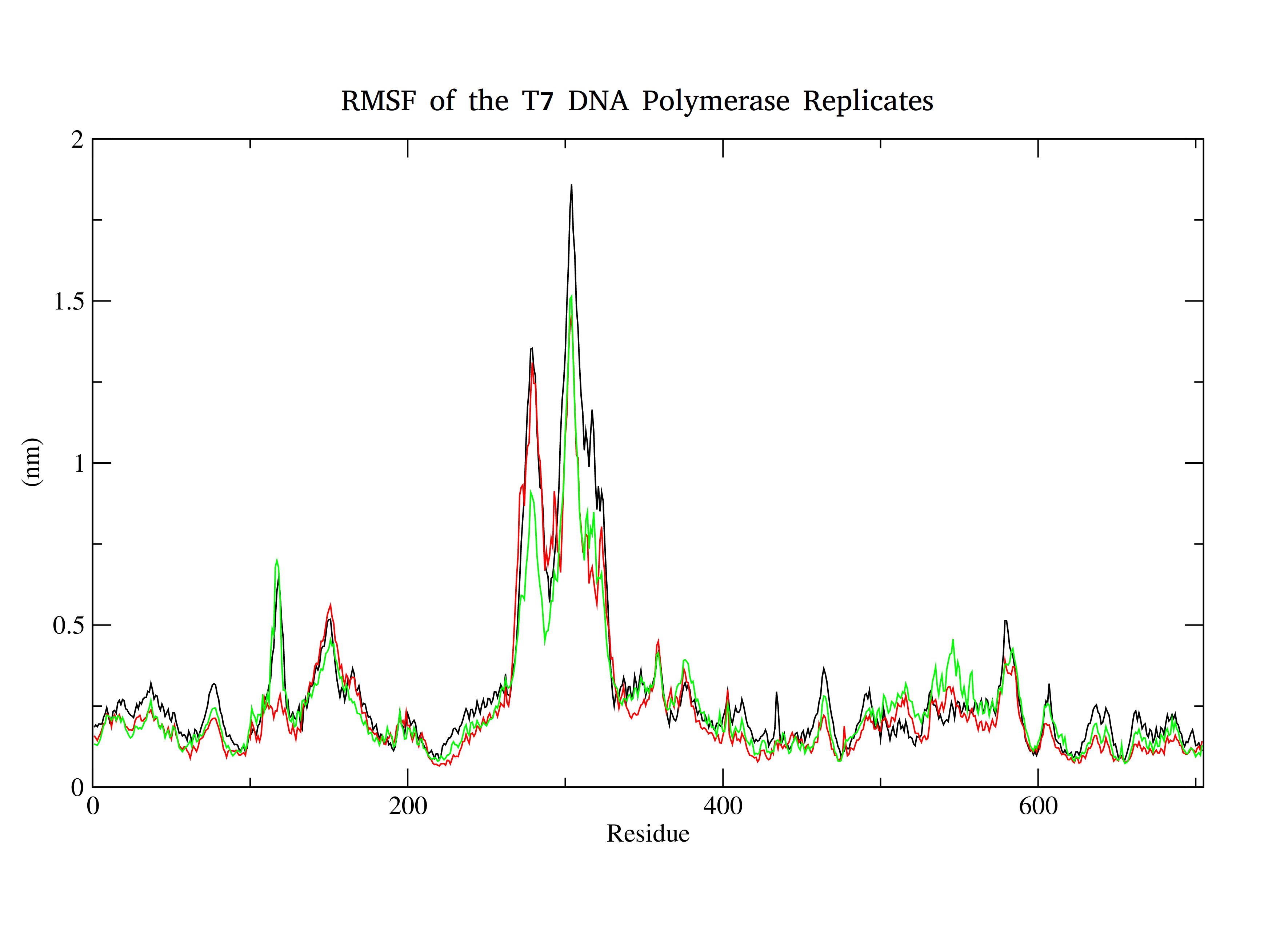

Supplement: Supplementary file 1 — (PNG 269 kb) [file 894_2018_3671_MOESM1_ESM.png]

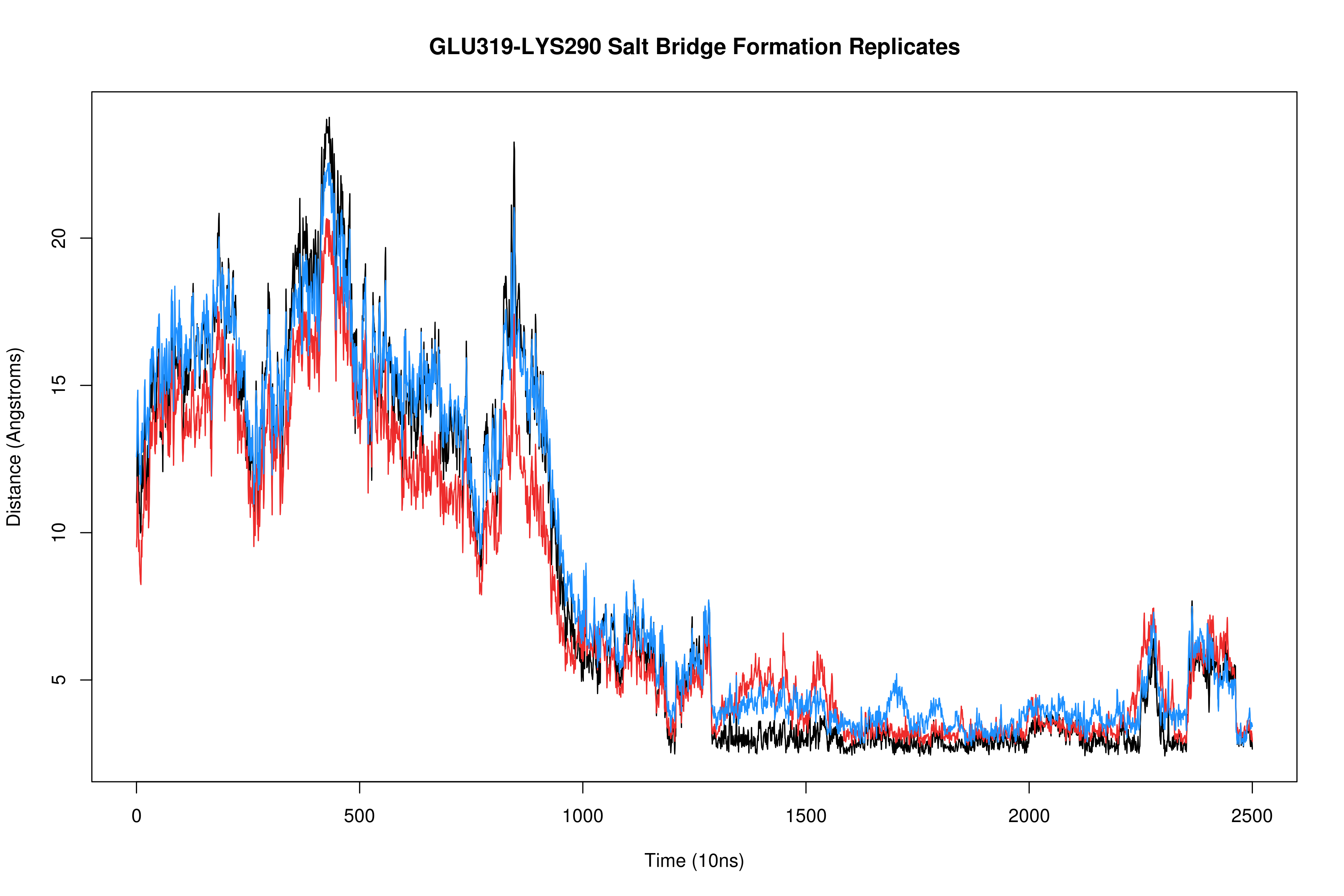

Supplement: Supplementary file 2 — (PNG 626 kb) [file 894_2018_3671_MOESM2_ESM.png]

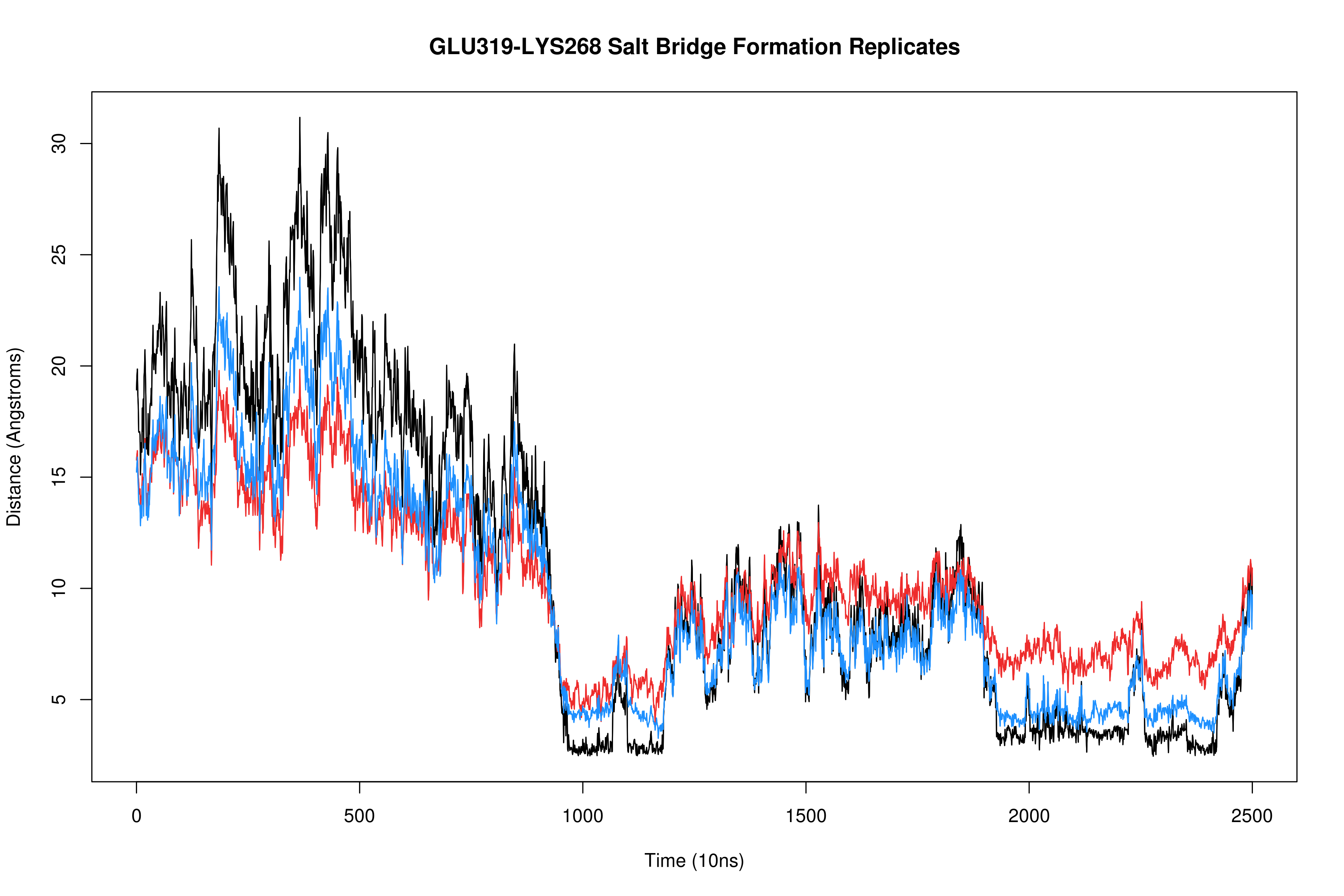

Supplement: Supplementary file 3 — (PNG 676 kb) [file 894_2018_3671_MOESM3_ESM.png]

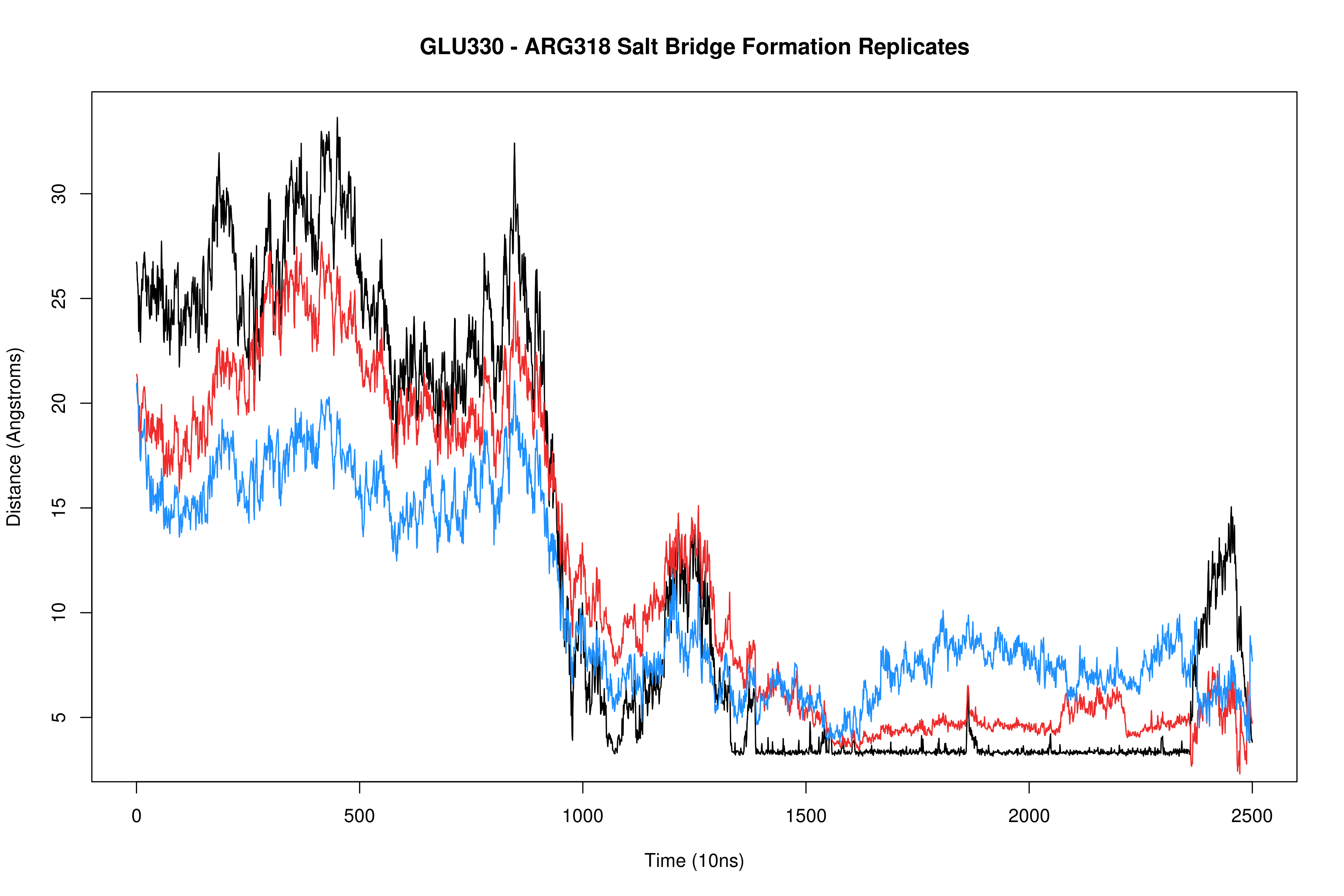

Supplement: Supplementary file 4 — (PNG 631 kb) [file 894_2018_3671_MOESM4_ESM.png]

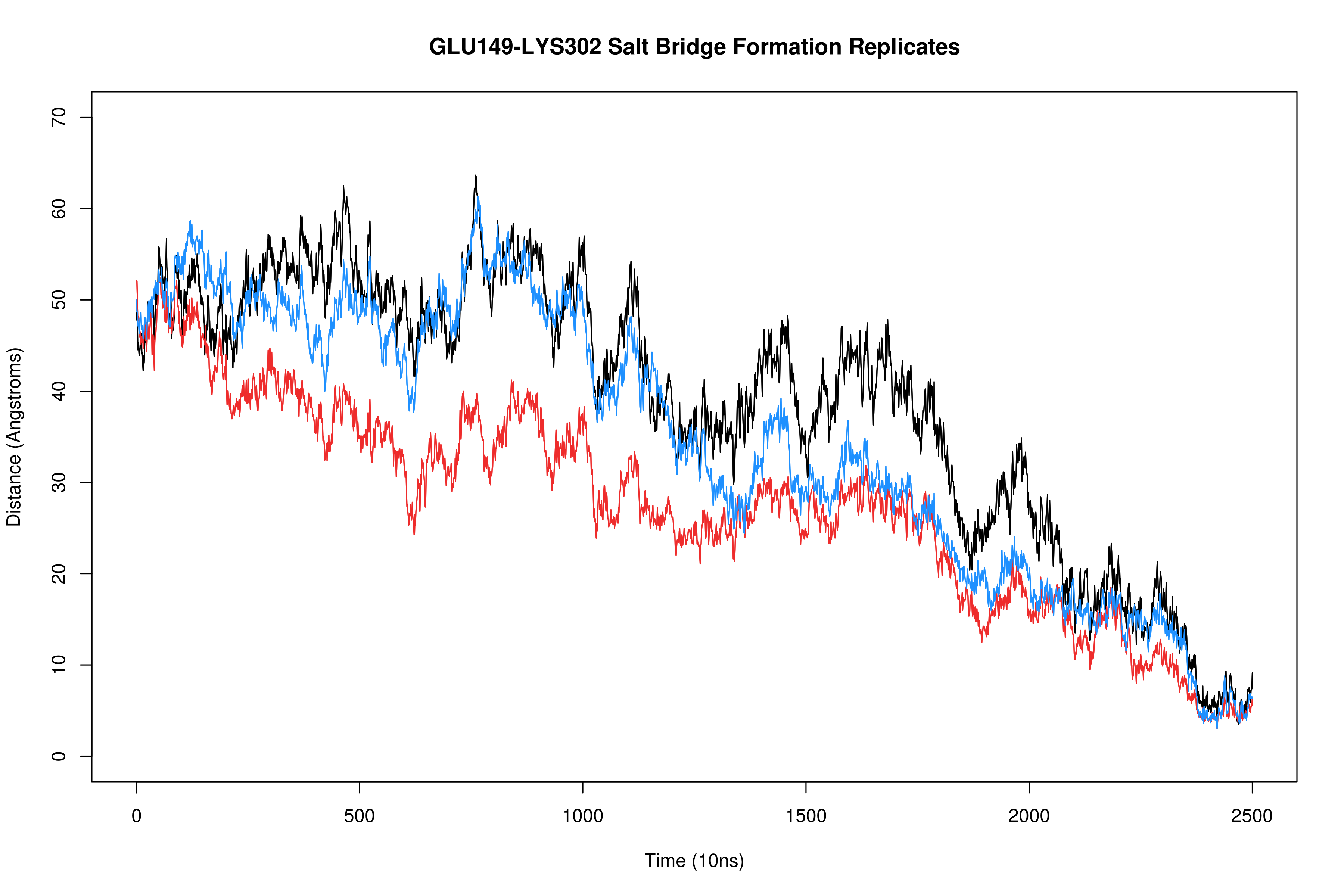

Supplement: Supplementary file 5 — (PNG 613 kb) [file 894_2018_3671_MOESM5_ESM.png]

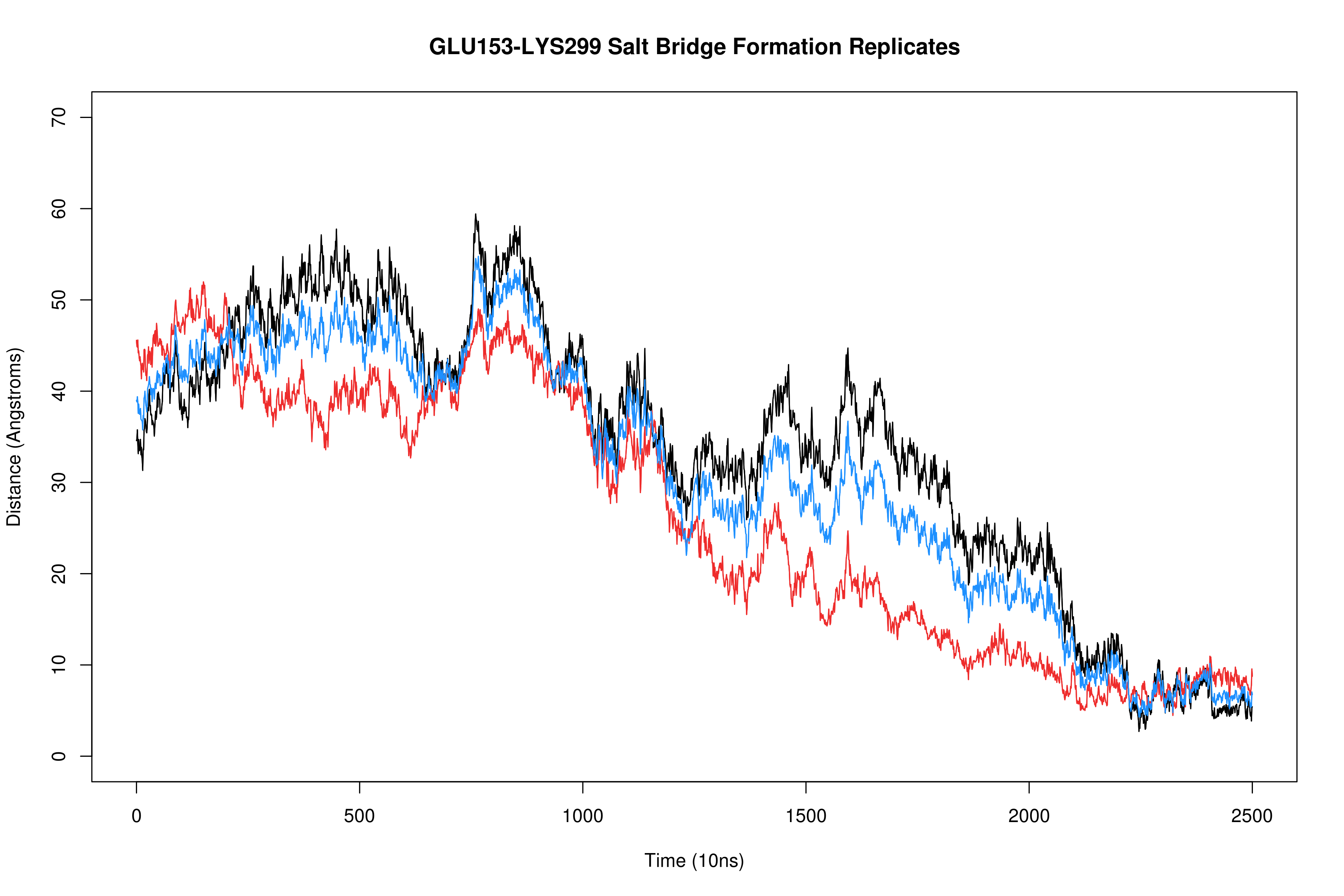

Supplement: Supplementary file 6 — (PNG 576 kb) [file 894_2018_3671_MOESM6_ESM.png]

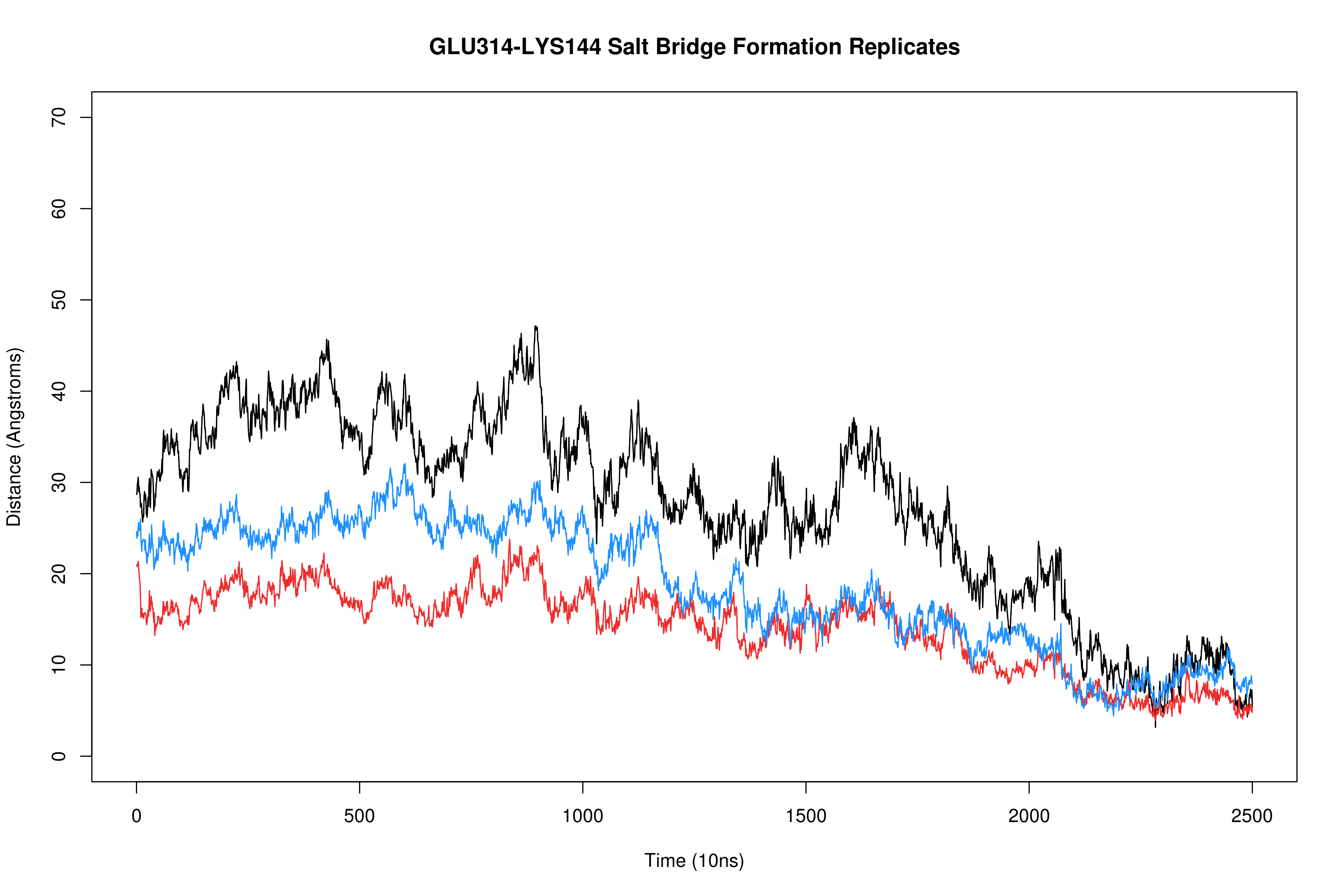

Supplement: Supplementary file 7 — (PNG 523 kb) [file 894_2018_3671_MOESM7_ESM.png]

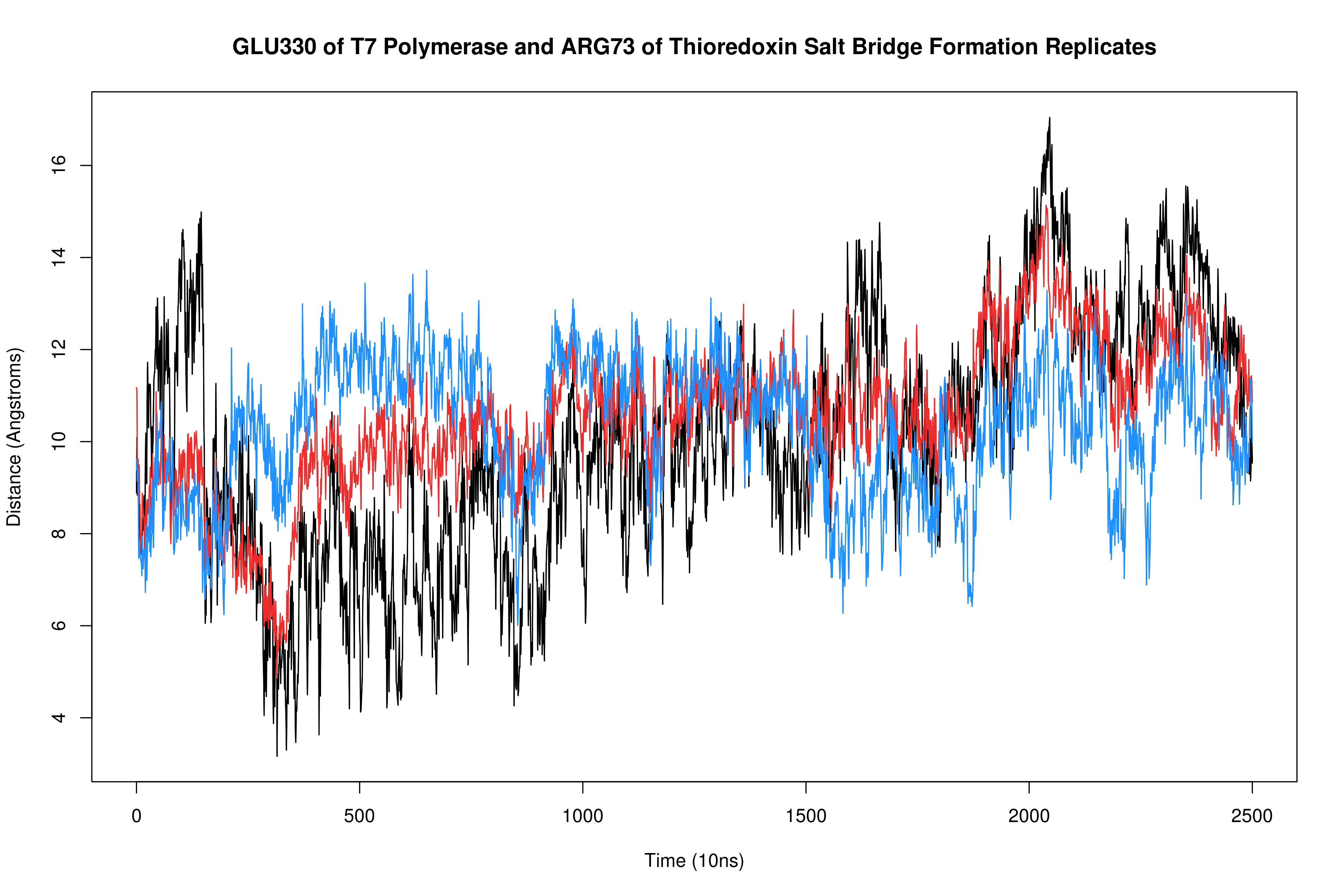

Supplement: Supplementary file 8 — (PNG 904 kb) [file 894_2018_3671_MOESM8_ESM.png]

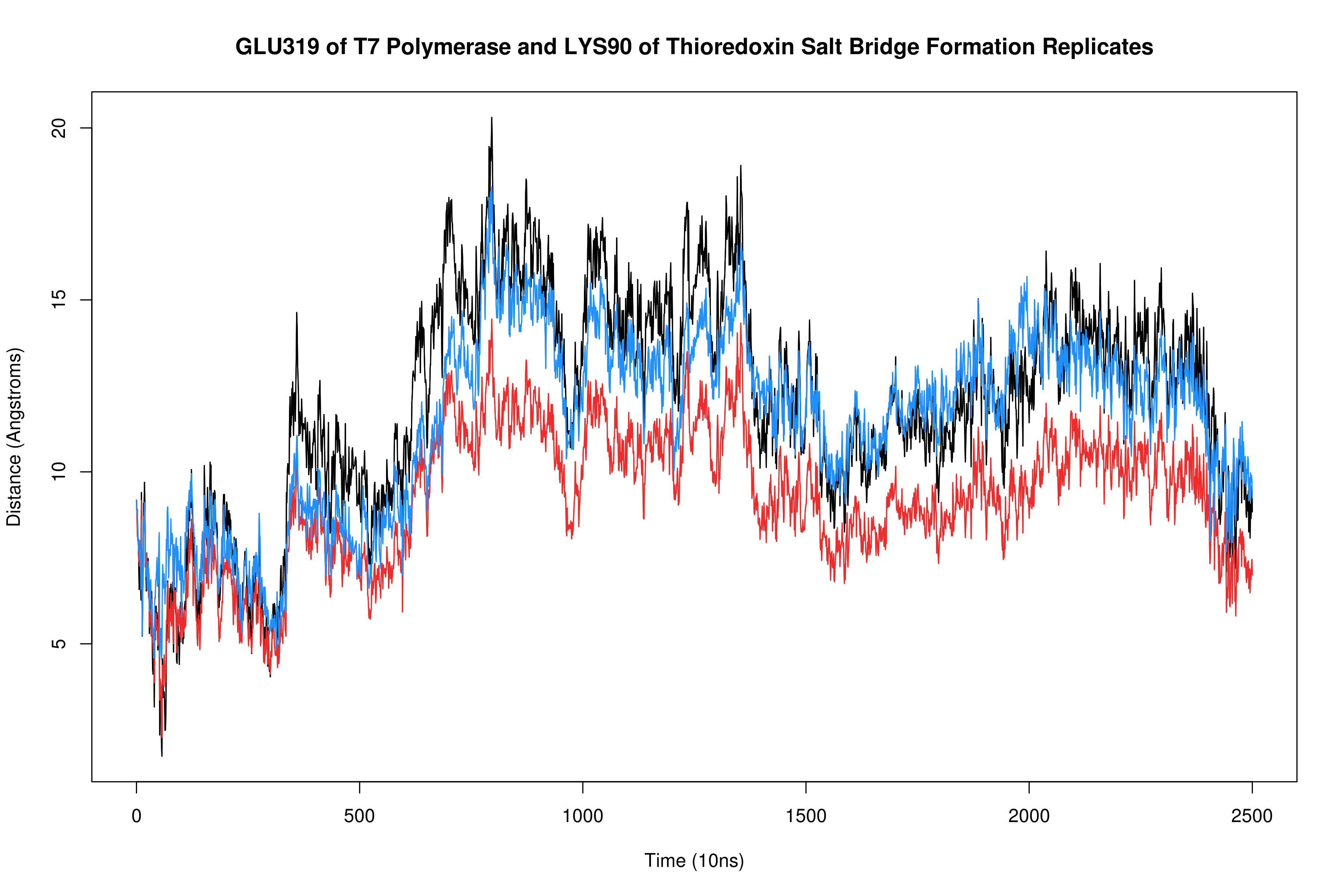

Supplement: Supplementary file 9 — (PNG 786 kb) [file 894_2018_3671_MOESM9_ESM.png]

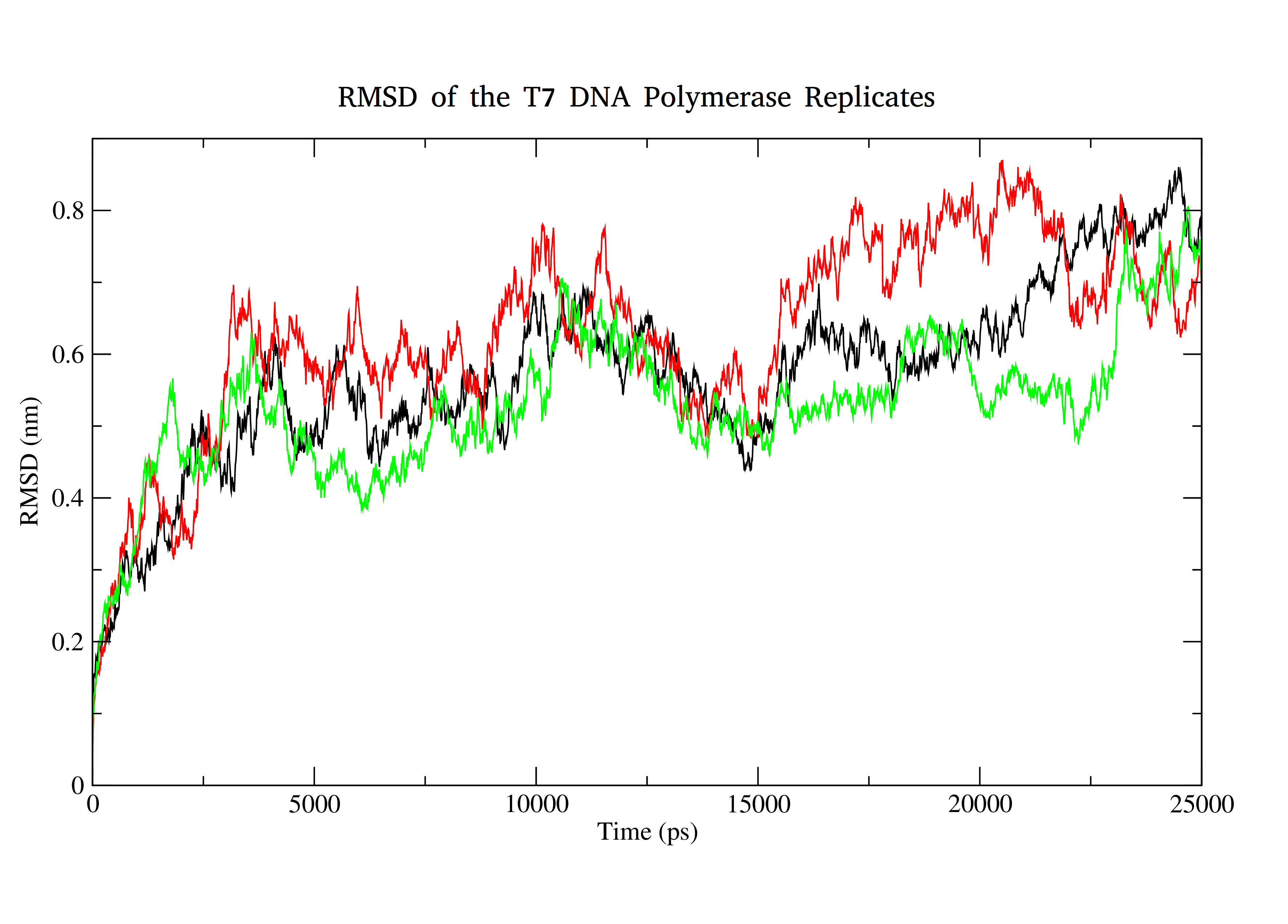

Supplement: Supplementary file 10 — (PNG 311 kb) [file 894_2018_3671_MOESM10_ESM.png]

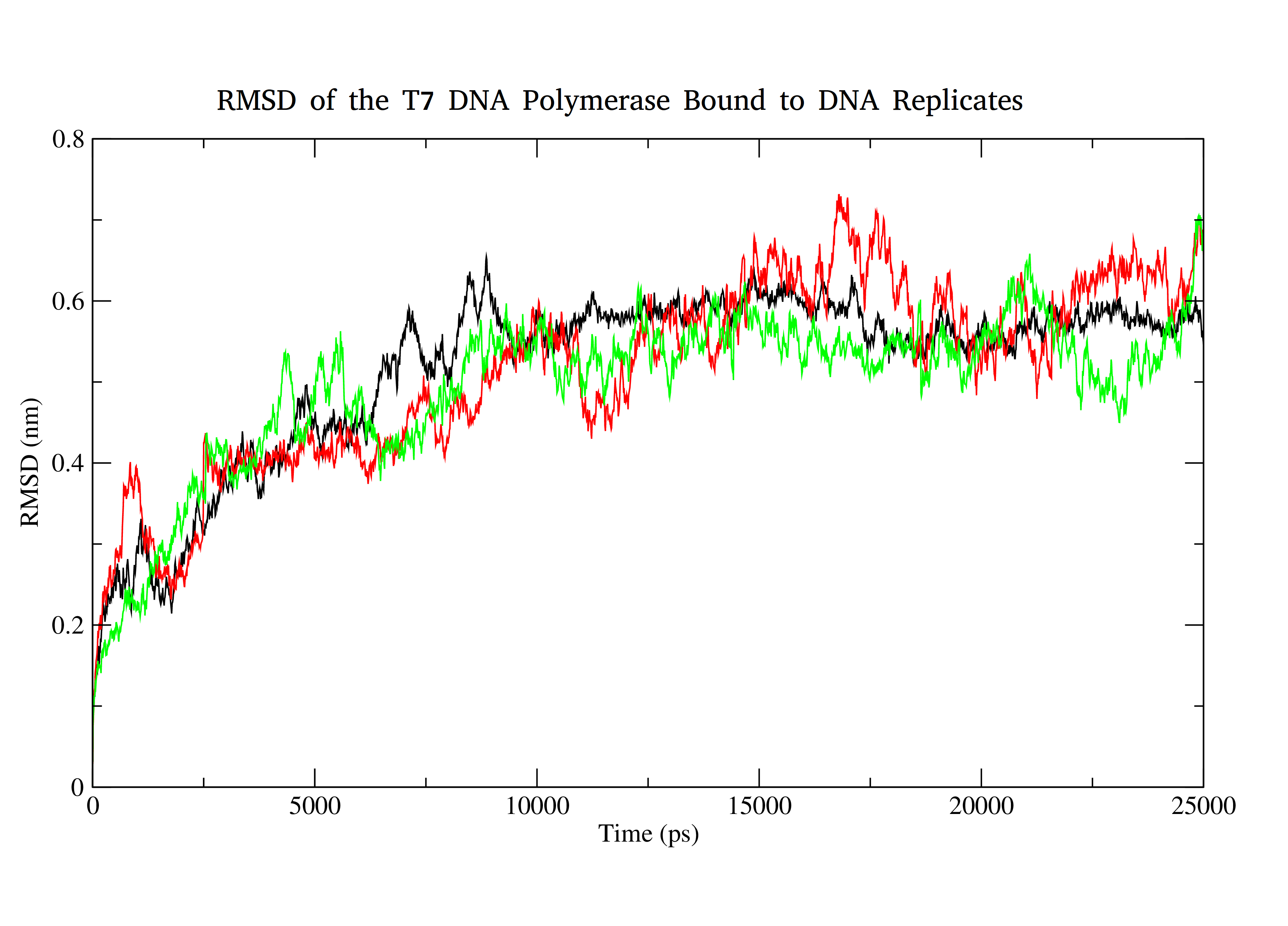

Supplement: Supplementary file 11 — (PNG 301 kb) [file 894_2018_3671_MOESM11_ESM.png]

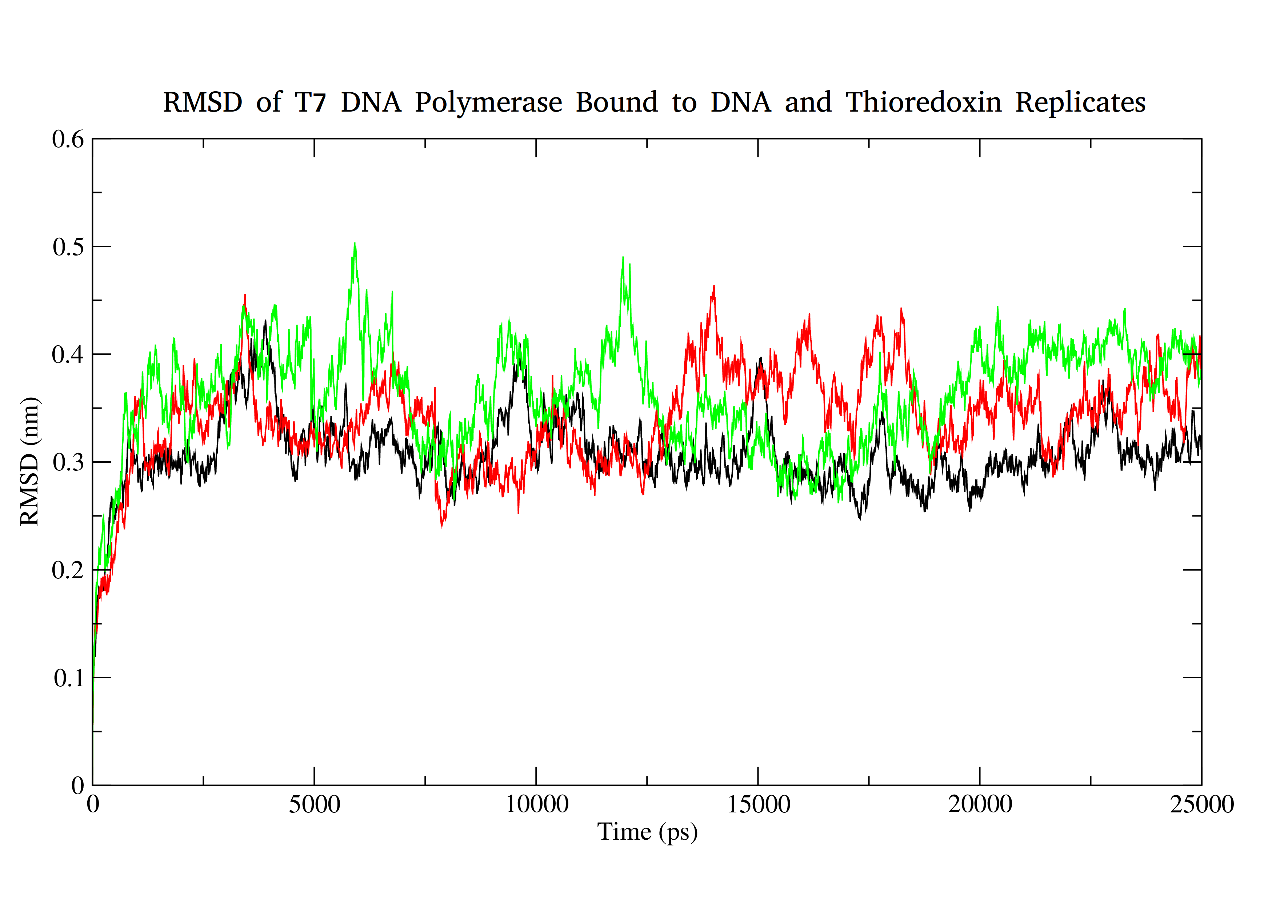

Supplement: Supplementary file 12 — (PNG 301 kb) [file 894_2018_3671_MOESM12_ESM.png]

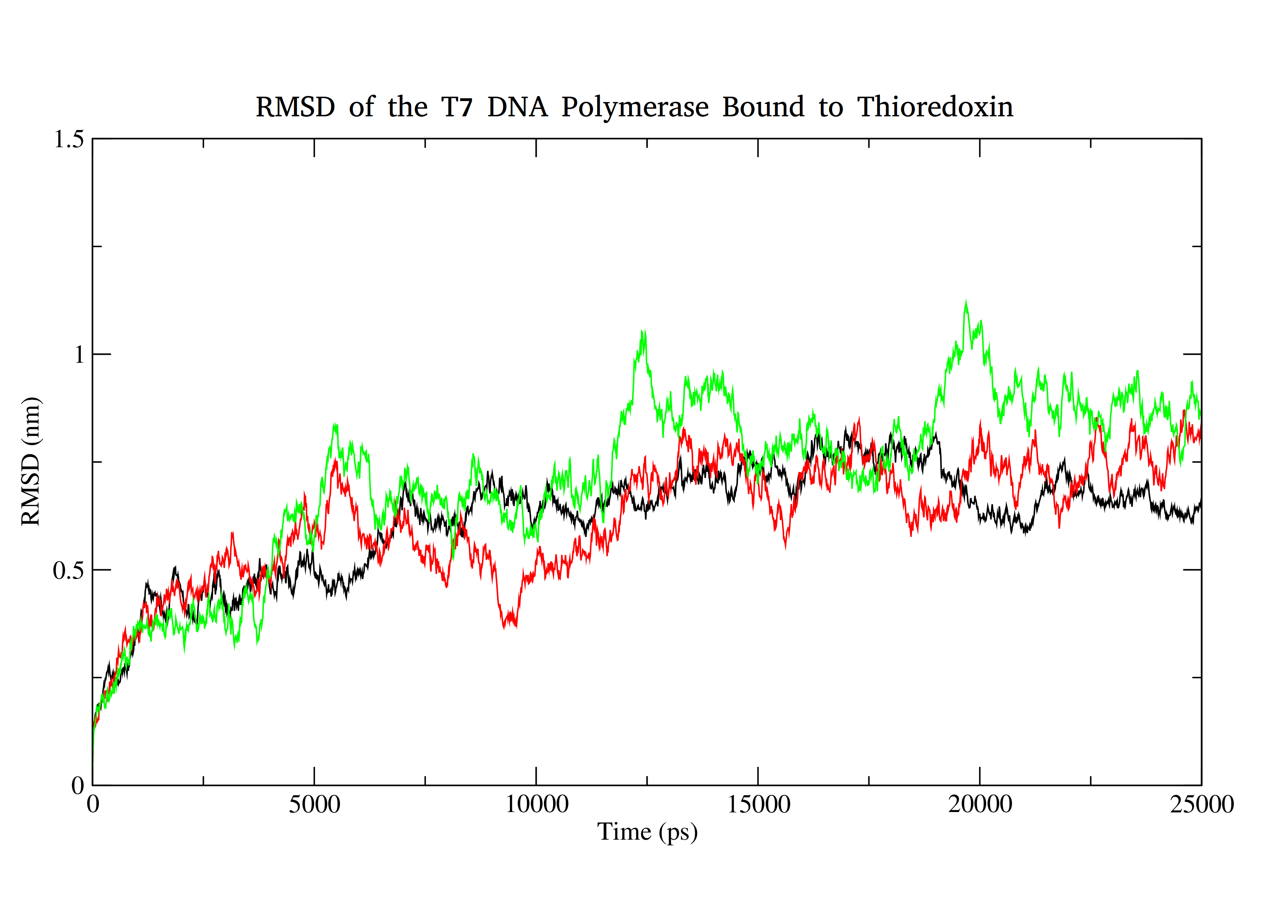

Supplement: Supplementary file 13 — (PNG 303 kb) [file 894_2018_3671_MOESM13_ESM.png]

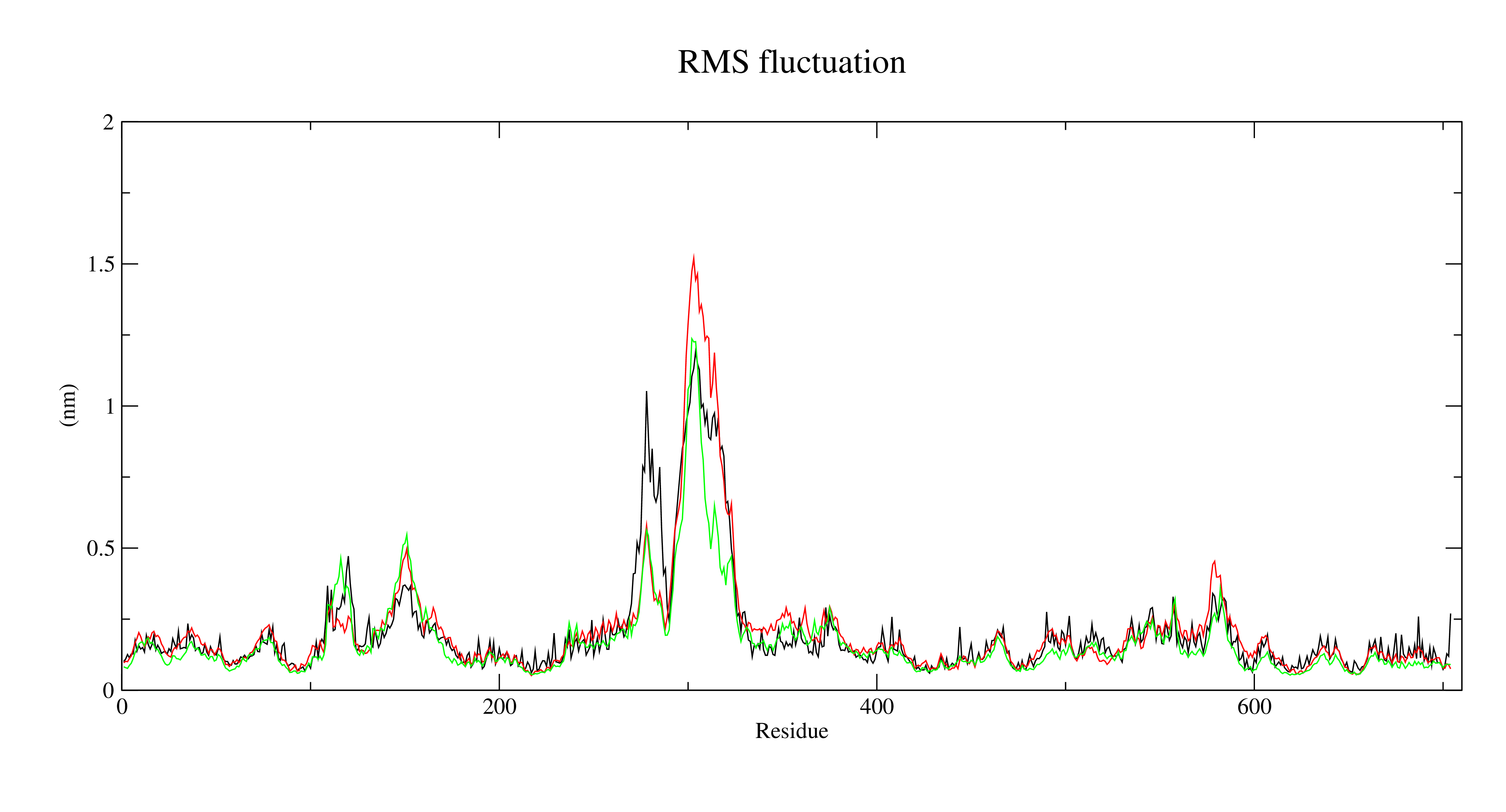

Supplement: Supplementary file 14 — (PNG 290 kb) [file 894_2018_3671_MOESM14_ESM.png]

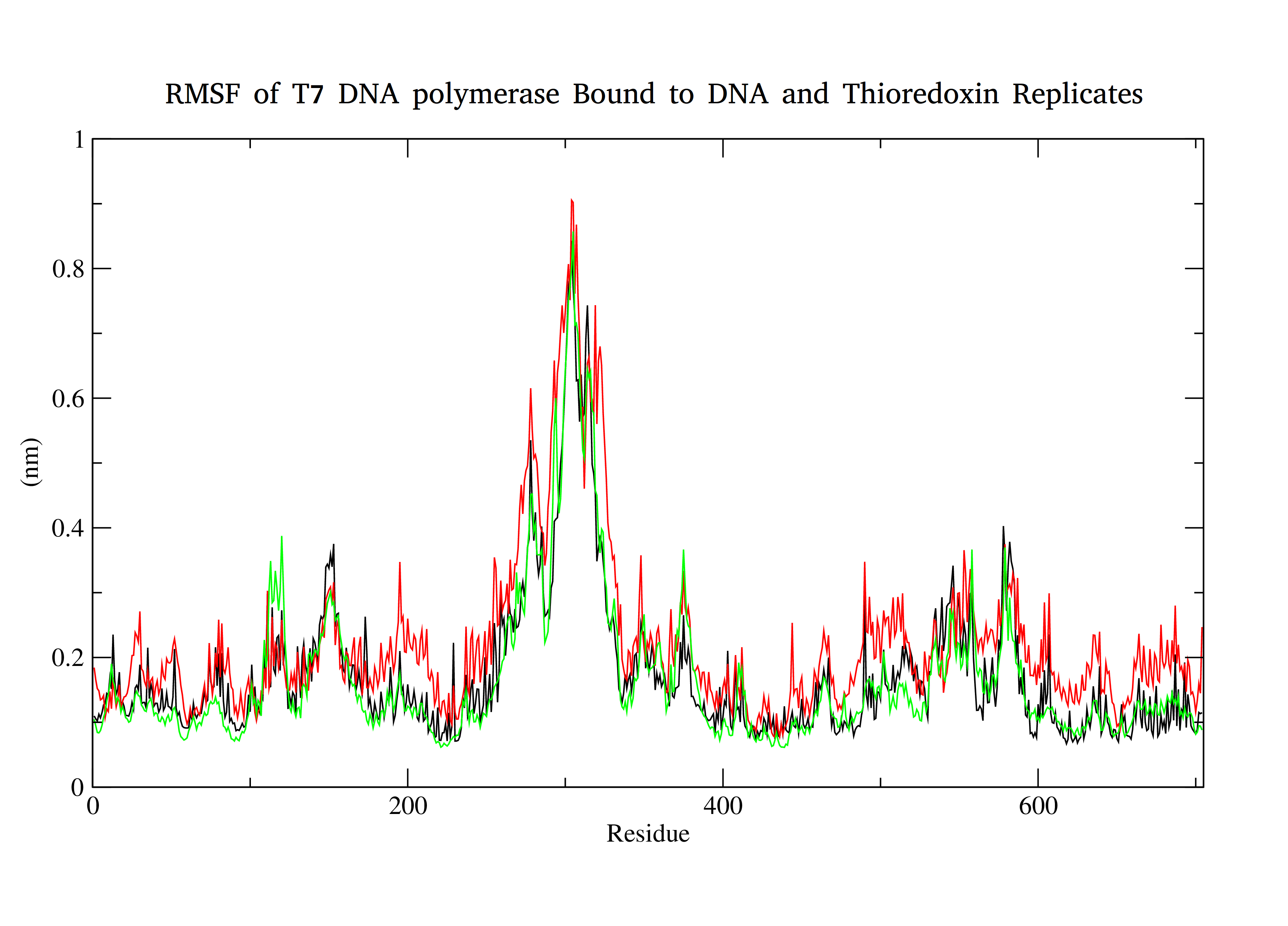

Supplement: Supplementary file 15 — (PNG 322 kb) [file 894_2018_3671_MOESM15_ESM.png]

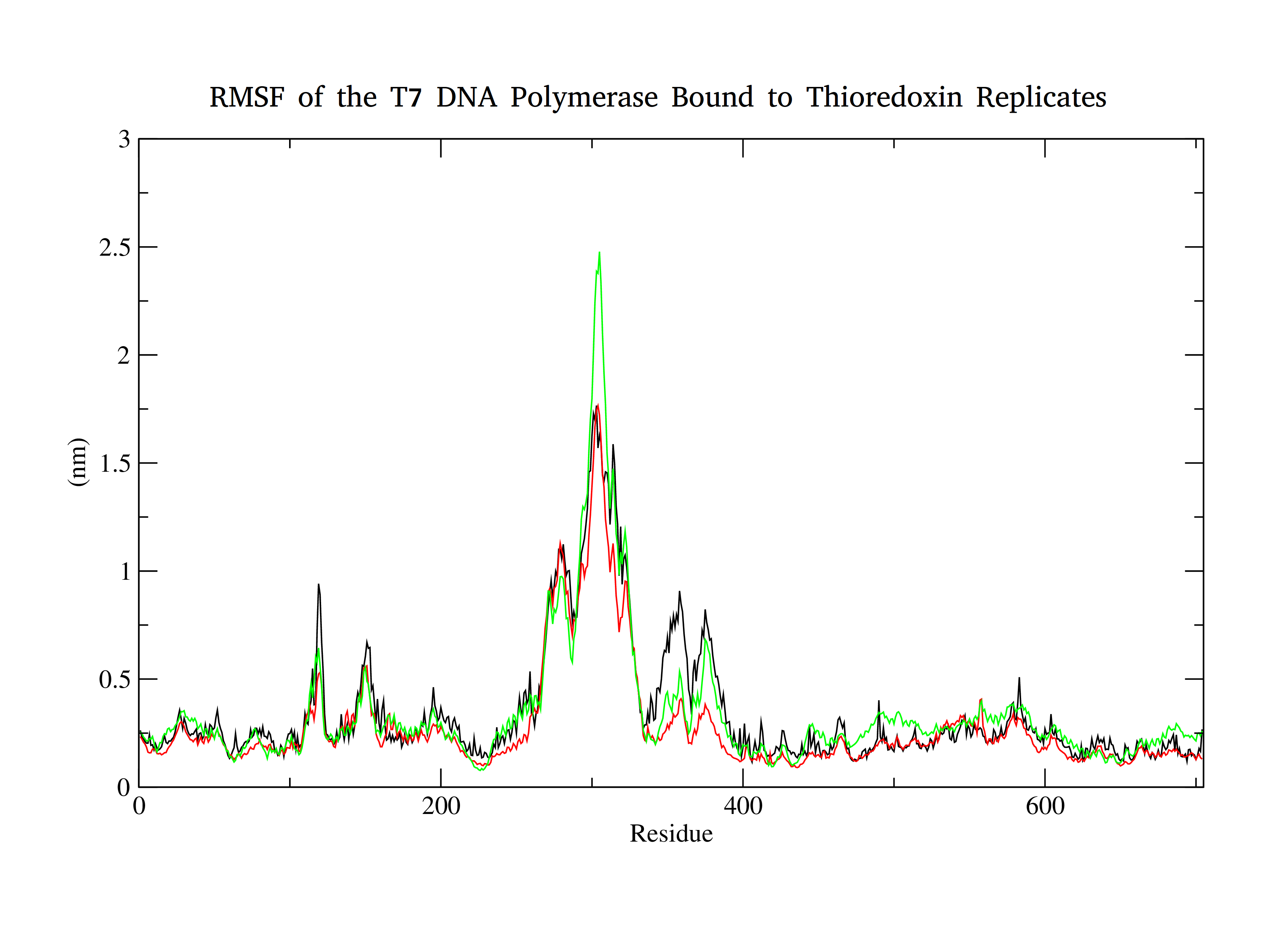

Supplement: Supplementary file 16 — (PNG 256 kb) [file 894_2018_3671_MOESM16_ESM.png]
